# Convergent Loss of Chemoreceptors across Independent Origins of Slave-Making in Ants

**DOI:** 10.1093/molbev/msab305

**Published:** 2021-10-20

**Authors:** Evelien Jongepier, Alice Séguret, Anton Labutin, Barbara Feldmeyer, Claudia Gstöttl, Susanne Foitzik, Jürgen Heinze, Erich Bornberg-Bauer

**Affiliations:** 1 Institute for Evolution and Biodiversity, Westfälische Wilhelms University, Münster, Germany; 2 Institute for Biodiversity and Ecosystem Dynamics, University of Amsterdam, Amsterdam, The Netherlands; 3 Molecular Ecology Group, Biodiversity and Climate Research Centre, Frankfurt am Main, Germany; 4 Institute for Zoology, University of Regensburg, Regensburg, Germany; 5 Institute of Organismic and Molecular Evolution, Johannes Gutenberg University, Mainz, Germany

**Keywords:** convergent gene loss, chemoreceptors, social parasitism, slave-making ants

## Abstract

The evolution of an obligate parasitic lifestyle often leads to the reduction of morphological and physiological traits, which may be accompanied by loss of genes and functions. Slave-making ants are social parasites that exploit the work force of closely related ant species for social behaviors such as brood care and foraging. Recent divergence between these social parasites and their hosts enables comparative studies of gene family evolution. We sequenced the genomes of eight ant species, representing three independent origins of ant slavery. During the evolution of eusociality, chemoreceptor genes multiplied due to the importance of chemical communication in insect societies. We investigated the evolutionary fate of these chemoreceptors and found that slave-making ant genomes harbored only half as many gustatory receptors as their hosts’, potentially mirroring the outsourcing of foraging tasks to host workers. In addition, parasites had fewer odorant receptors and their loss shows striking patterns of convergence across independent origins of parasitism, in particular in orthologs often implicated in sociality like the 9-exon odorant receptors. These convergent losses represent a rare case of convergent molecular evolution at the level of individual genes. Thus, evolution can operate in a way that is both repeatable and reversible when independent ant lineages lose important social traits during the transition to a parasitic lifestyle.

## Introduction

Gene losses are pervasive throughout the animal kingdom (Guijarro-Clarke et al. 2020) and may constitute a frequent evolutionary response (Albalat and Cañestro 2016). Indeed, the less-is-more hypothesis (Olson 1999) proposes that loss of gene function may occur often and spread through populations, as a change in environment or behavior can render certain genes nonessential. Additionally, gene loss can open alternative evolutionary trajectories through modifications of genetic network structure, and can even lead to higher fitness in certain environments (Helsen et al. 2020). Among the reported cases of convergent gene loss across independent taxonomic lineages are the loss of an enzyme required for vitamin C biosynthesis in several vertebrate lineages (Drouin et al. 2011) and the convergent loss of *Paraoxonase 1* in several marine mammals (Meyer et al. 2018). The latter is likely due to parallel shifts in lipid metabolism in marine ancestors. Such convergent patterns of gene loss accompanying shifts in environment or behavior suggest that losses may potentially be adaptive. Conversely, gene losses may reveal which genes are essential in specific environments and which genomic changes underpinned major evolutionary shifts.

One such evolutionary shift and arguably the most common major shift in life history strategy (Poulin and Randawa 2013) is the evolution of parasitism. Since parasites exploit their hosts, they often lose the capacity for independent resource acquisition (Baxter et al. 2010; Kirkness et al. 2010; Mitreva et al. 2011; Sun et al. 2018). Several scenarios may explain the possible benefits of gene losses accompanying the loss of traits during such a transition to parasitism. First, gene losses may release some epistatic constraints because other genes may become free to adapt if those which constrain their activity are lost (Helsen et al. 2020). Second, losses themselves may change a trait such that a parasite’s fitness is increased, for example, because the lost trait itself was detrimental (but not prohibitive) for exploiting the host (Sokurenko et al. 1999). Finally, losses of genes underlying traits that are dispensable in a new environment (e.g., the shift to parasitic lifestyle) may be favored by selection for a reduction in metabolic costs (Albalat and Cañestro 2016).

Although the benefits of acquired genes and the utilization of existing or duplicated genes (Kondrashov 2012; Qian and Zhang 2014) for novel traits have been well studied through comparative evolutionary genomics and transcriptomics (Zhou et al. 2019; Whitelaw et al. 2020; Calla 2021), the benefits of gene losses are more difficult to verify. The study of convergent gene loss patterns may represent a first step in this direction, as such convergence provides insights into potential adaptive benefits of gene losses. However, technical and phylogenetic limitations make it difficult to elucidate the molecular patterns underlying parallel loss of morphological or behavioral traits. Lost genes can no longer be analyzed and host–parasite systems that are amenable to experimental or computational investigations typically consist of phylogenetically distant species. To overcome these limitations, we investigated genomic changes underlying the transition to social parasitism in slave-making ants, an iconic group of social insects already mentioned by Darwin (1859). Although all ants are ancestrally eusocial, strongly relying on chemical signals to organize tasks within and outside the social colony (Hölldobler 1995), some species have secondarily lost key social traits, such as the worker caste in inquiline social parasites, or, to a lesser extent, foraging and nursing behaviors in workers of slave-making ants (Buschinger 1986).

Slave-making ants are obligate social parasites that completely rely on workers of closely related host species for brood care, nest defense, foraging, and other nest maintenance tasks, all of which demand chemical communication mediated by olfaction. Furthermore, worker reproduction, which is unusual in ant colonies in the presence of a queen, is prevalent in slave-making ants. This suggests that slave-making ant workers may have lost their ability to perceive and respond to queen pheromones (Heinze 1996). We expect gene losses, specifically the loss of chemosensory genes which radiated during social evolution in ants, to act as an important mechanism underpinning the loss of social behavior in slave-making ants.

The myrmicine *Formicoxenus* species group (formerly Formicoxenini; Blaimer et al. 2018) is a hot spot for the evolution of social parasitism, with at least five independent origins of slave-making (Beibl et al. 2005; Feldmeyer et al. 2017; Prebus 2017). Like most social parasites (Emery 1909), these slave-making ants from the genera *Temnothorax* and *Harpagoxenus* exploit closely related nonparasitic species of *Temnothorax* and *Leptothorax* (Hölldobler and Wilson 1990), although not all members of these taxa are parasitized. The close relatedness between hosts and slave-making ants and the associated similar genomic architecture render them ideal systems to study co-evolutionary arms races (Foitzik et al. 2001; Feldmeyer et al. 2017; Alleman et al. 2018). Furthermore, slave-making ants and hosts share the same nest and thus have very similar ecological and physiological requirements. This reduces possible confounding factors. In addition, closely related nonhost, nonparasitic ant species resemble the most likely ancestral state and thus serve as convenient natural controls in genomic comparisons.

**Fig. 1.**
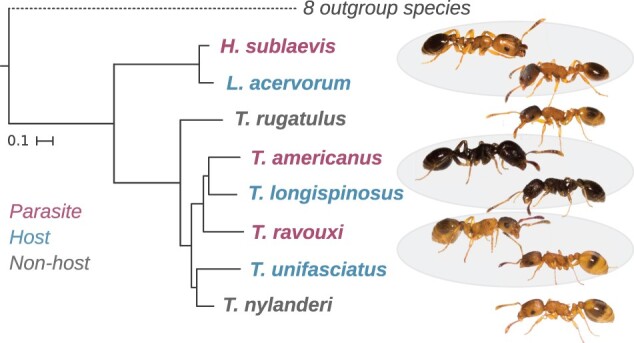
Phylogeny of the focal species. Gray ellipses highlight the three independent slave-making ant–host pairs. Scale represents the number of substitutions per site. Outgroup ant species are listed in supplementary table S11, Supplementary Material online. The dashed line to the eight outgroup species is not to scale. Tree based on Prebus (2017). Pictures by Barbara Feldmeyer.

In this study, we concentrate on reconstructing the evolutionary history of chemoreceptor (odorant and gustatory) genes and investigate convergent evolutionary patterns across the independent origins of slave-making in ants. We ask: 1) what are the defining changes in chemosensory gene repertoire underlying the repeated evolution of slave-making in ants, 2) are these changes convergent across multiple origins of slave-making in ants, and 3) if so, are these convergent changes (e.g., losses) more frequent than expected by chance? The convergent contraction of these gene families would indicate parallel evolutionary changes during a shift toward a parasitic lifestyle. To address these questions, we analyze high-quality genome assemblies of eight ant species based on PacBio long read sequencing technology. These include the genomes of three slave-making ants, representing three independent and distant origins of social parasitism within the *Formicoxenus* group, three hosts that are the primary hosts of the sequenced slave-making ant species, and two nonhost sister species (see fig. 1).

## Results

### Eight Newly Sequenced Ant Genomes Harbor Many Chemosensory Receptor Genes

Using a combination of newly sequenced PacBio reads and Illumina short read genomic data for three slave-making ant species (*Harpagoxenus sublaevis*, *Temnothorax ravouxi—*formerly *Myrmoxenus ravouxi*, *T. americanus—*formerly *Protomognathus americanus*), three host species (*Leptothorax acervorum*, *T. unifasciatus*, *T. longispinosus*), and two nonhost sister species (*T. rugatulus*, *T. nylanderi*), we assembled eight novel genomes across three independent origins of slave-making (see Materials and Methods for details on sample collection, sequencing, and genome assembly). Several genome assembly strategies were explored, resulting in eight highly complete (complete BUSCOs: mean ± SD=98.1%±1.2) and comparable genomes (table 1). We manually annotated 3,718 chemosensory receptor genes across all eight species, including 3,007 odorant receptor genes (*Or*s) and 711 gustatory receptor genes (*Gr*s).

**Table 1.** Genome Assembly Statistics.

	*Harpagoxenus sublaevis*	*Leptothorax acervorum*	*Temnothorax ravouxi*	*Temnothorax unifasciatus*	*Temnothorax americanus*	*Temnothorax longispinosus*	*Temnothorax rugatulus*	*Temnothorax nylanderi*
Estimated genome size (Mb)	357	371	370	313	307	310	335	305
Assembly length (Mb)	342	345	362	314	280	298	356	313
GC (%)	39.00	38.67	38.70	38.81	39.20	38.93	39.31	39.06
Number of contigs	2,750	1,269	4,380	2,496	1,229	3,430	2,079	1,555
Largest contig (kb)	4,364	6,339	1,282	2,901	4,381	1,041	4,513	4,914
N50 (kb)	391	730	129	360	603	156	451	619
L50	176	104	751	196	121	541	155	111
Complete BUSCOs (%)	98.1	98.3	95.7	98.2	98.2	95.6	98.4	98.5
Duplicated BUSCOs (%)	1.4	1.6	7.7	2.8	1.3	1.8	2.3	2.4
Illumina mapping rate (%)	97.87	97.24	96.76	96.32	97.22	95.77	95.98	97.33
PacBio mapping rate (%)	91.89	91.59	96.28	91.24	97.34	92.11	94.08	93.27

### Extensive Chemosensory Receptor Losses in Slave-Making Ants, Modest Expansions in Hosts

**Fig. 2.**
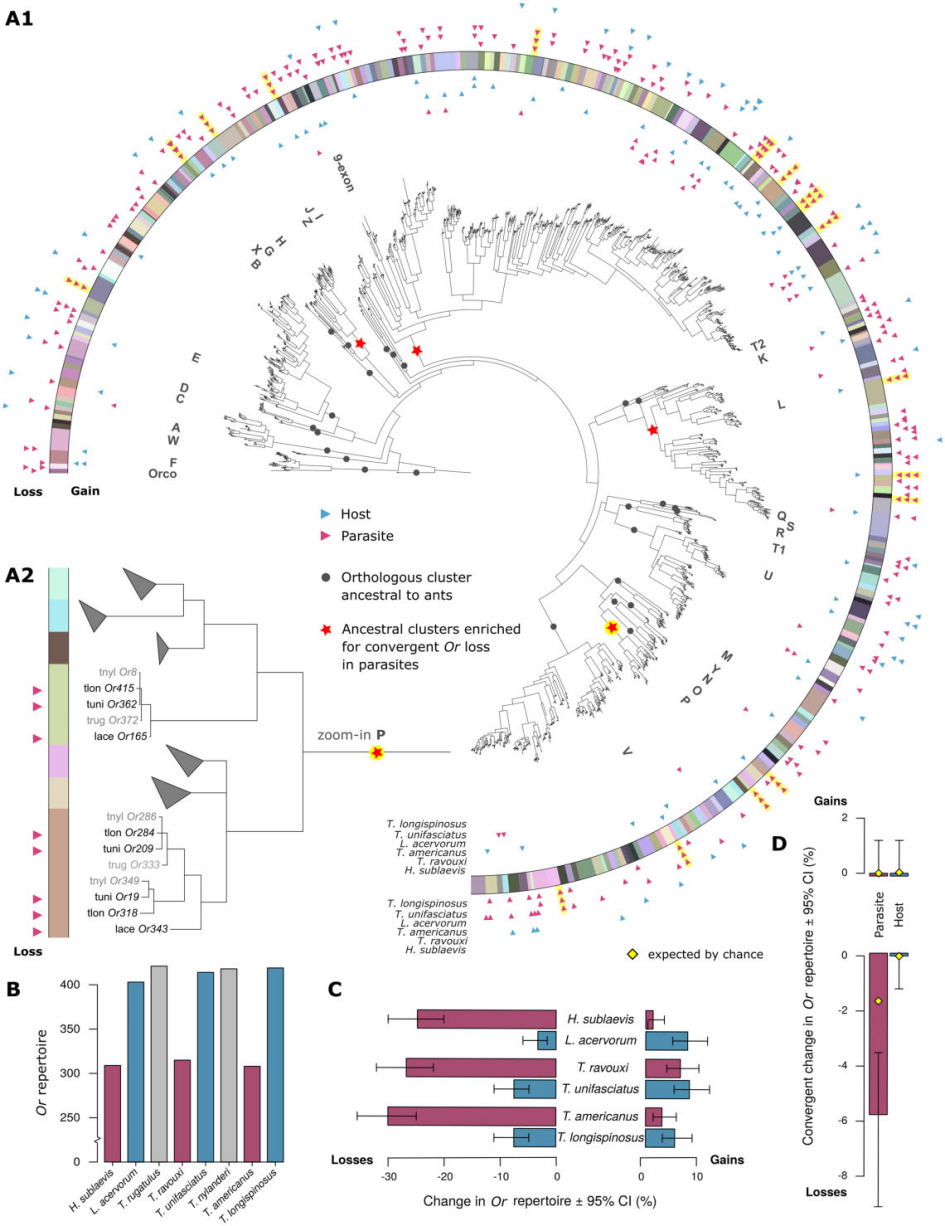
Distribution of odorant receptor gene (*Or*) gains and losses across species and orthologous clusters. (*A1*) Phylogenetic clustering of *Or*s across three slave-making ant species and their hosts. Species-specific expansions were collapsed for visualization purposes. Orthologous cluster assignments are displayed through colors in the outer ring. Subfamilies identified in previous studies as being ancestral to the ants, bees, and wasps are represented by dark circles and corresponding letters throughout the tree. We found that four of these ancestral clusters were enriched for convergent *Or* loss in parasites, marked by the red stars in the tree. Triangles on the inner side of the colored ring show the distribution of gene gains across orthologous clusters and triangles on the outer side of the ring show the distribution of gene losses. Convergent losses are highlighted with yellow shading of triangles signifying loss. Species names at the end of the ring indicate the species in which the gains or losses occurred, with red triangles showing gains/losses in slave-making ant (parasite) species and blue triangles showing gains/losses in host species. (*A2*) Zoom-in on ancestral cluster P, containing seven orthologous clusters. Five clusters, in which no genes were gained or lost, were collapsed. In the remaining two clusters, gene copies were convergently lost in all three slave-making ant species. Red triangles indicate a gene loss, as exhibited by the presence of a copy in a host species, but not in its respective parasite species. (*B*) Number of *Or* copies in each focal species. Bars are color-coded according to lifestyle of the species as in the following graphs, with red representing parasite species, blue host species, and gray outgroup species. (*C*) Relative change in *Or* repertoire in slave-making ants compared with their respective hosts, and in hosts compared with their respective parasites. The 95% CIs were computed using the R stats: exact binomial test. (*D*) Number of cases of convergent gain (top) or loss (bottom) of *Or*s in slave-making ants and in hosts. An event (gain or loss) was defined as convergent if it occurred in all three parasite or host species within an orthologous cluster. The yellow diamonds represent the null-probability, set to the product of the marginal probabilities of loss for each parasite, that is, the probability that convergent loss occurred by chance in each of the three parasites.

Slave-making ants exhibited smaller *Or* repertoires than all host and nonhost species (fig. 2*B* and table 2). Slave-making ant genomes contained on average 311 *Or*s (range 308–315), which did not vary significantly among slave-making ant species (table 2). In contrast, each host and nonhost genome harbored more than 400 *Or*s (range 403–421, supplementary table S14, Supplementary Material online), again with *Or* numbers similar across all host species (table 2).

**Table 2.** Chemoreceptor Repertoire Size and Turn-Over Statistics in Slave-Making Ants and Their Hosts.

	Stat	df	*P*_adj_
Comparison of *Or* repertoire size			
Slave-making ants versus hosts	20.10	2	0.002
Across slave-making ants	1.838	2	0.399
Across hosts	6.427	2	0.080
			
Comparison of *Gr* repertoire size			
Slave-making ants versus hosts	8.64	2	0.013
Across slave-making ants	1.927	2	0.382
Across hosts	6.261	2	0.087
			
	$\chi^{2}$	df	*P*_adj_
Comparison of number of *Or* losses			
Slave-making ants versus hosts	868.44	3	$2.200\times 10^{-16}$
*L. acervorum—H. sublaevis*	58.34	1	$6.633\times 10^{-14}$
*T. unifasciatus—T. ravouxi*	41.81	1	$1.007\times 10^{-10}$
*T. longispinosus—T. americanus*	49.48	1	$3.014\times 10^{-12}$
			
Comparison of number of *Gr* losses			
Slave-making ants versus hosts	>999.99	3	$2.200\times 10^{-16}$
*L. acervorum—H. sublaevis*	52.65	1	$1.196\times 10^{-12}$
*T. unifasciatus—T. ravouxi*	25.24	1	$5.076\times 10^{-7}$
*T. longispinosus—T. americanus*	29.59	1	$7.997\times 10^{-8}$
			
Comparison of number of *Or* expansions			
Slave-making ants versus hosts	21.34	3	$8.949\times 10^{-05}$
*L. acervorum—H. sublaevis*	12.55	1	0.001
*T. unifasciatus—T. ravouxi*	0.40	1	0.527
*T. longispinosus—T. americanus*	1.50	1	0.331
			
Comparison of number of *Gr* expansions			
Slave-making ants versus hosts	35.32	3	$1.045\times 10^{-7}$
*L. acervorum—H. sublaevis*	14.45	1	$4.317\times 10^{-4}$
*T. unifasciatus—T. ravouxi*	6.299	1	0.018
*T. longispinosus—T. americanus*	1.565	1	0.211

**Fig. 3.**
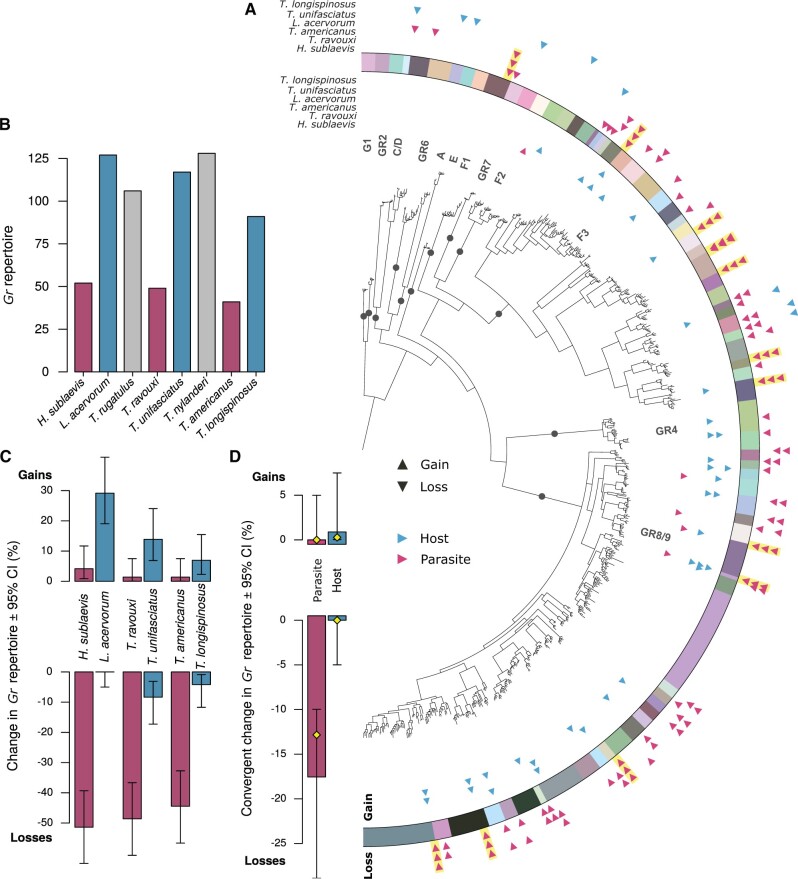
Distribution of gustatory receptor gene (*Gr*) gains and losses across species and orthologous clusters. (*A*) Phylogenetic clustering of *Gr*s across three slave-making ant species and their hosts. The gene tree was constructed using the same methods described in figure 2. Orthologous cluster assignment is displayed through colors in the outer ring. Subfamilies identified in previous studies as being ancestral to the ants, bees, and wasps are represented by dark circles and corresponding letters throughout the tree. Triangles on the inner side of the colored ring show the distribution of gene gains across orthologous clusters and triangles on the outer side of the ring show the distribution of gene losses. Convergent losses are highlighted with yellow shading of triangles signifying loss. Species names at the top indicate the species in which the gains or losses occurred, with red triangles showing gains/losses in slave-making ant (parasite) species and blue triangles showing gains/losses in host species. (*B*) Number of *Gr* copies in each focal species. Bars are color-coded according to lifestyle of the species as in following graphs, with red representing parasite species, blue host species, and gray outgroup species. (*C*) Relative change in *Gr* repertoire in slave-making ants compared with their respective hosts, and in hosts compared with their respective parasites. The 95% CIs were computed using the R stats: exact binomial test. (*D*) Number of cases of convergent gain (top) or loss (bottom) of *Gr*s in slave-making ants and in hosts. An event (gain or loss) was defined as convergent if it occurred in all three parasite or host species within an orthologous cluster. The yellow diamonds represent the null-probability, set to the product of the marginal probabilities of loss for each parasite, that is, the probability that convergent loss occurred by chance in each of the three parasites.

Note.—Statistics represent three-sample (comparisons of repertoire size) or two-sample (comparisons of losses and expansions) test for equality of proportions with continuity correction. See figures 2*B*, 2*C*, 3*B*, and 3*C* for effect sizes. *L. acervorum*, *Leptothorax acervorum*; *H. sublaevis*, *Harpagoxenus sublaevis*; *T. unifasciatus*, *Temnothorax unifasciatus; T. longispinosus, Temnothorax longispinosus; T. americanus, Temnothorax americanus*.

Slave-making ants experienced more *Or* losses than their respective hosts across all three origins of parasitism (fig. 2*C* and table 2). The percentage of *Or*s that were lost in each slave-making lineage was very similar, ranging from 25.08% in *H. sublaevis* to 27.69% in *T. ravouxi* and 29.97% in *T. americanus* (fig. 2*C* and supplementary table S16, Supplementary Material online).

Slave-making ants also exhibited much smaller *Gr* repertoires than hosts and nonhost species (parasite range 41–52; host and nonhost range 91–128; fig. 3*B*; supplementary table S15, Supplementary Material online; 2), with *Gr* numbers similar across parasite genomes on the one hand and across host genomes on the other hand.

*Gr* loss was much more prevalent in slave-making ants than in their respective hosts (fig. 3*C* and table 2). The proportion of *Gr*s that were lost in slave-making ants was comparable across the three origins of parasitism, ranging from 55.56% in *H. sublaevis* to 47.22% in *T. ravouxi* and 44.44% in *T. americanus* (fig. 3*C* and supplementary table S16, Supplementary Material online). This indicates that the transition to parasitism is associated with extensive losses of both *Or*s and *Gr*s in the parasites’ genome.

Host species exhibited modest expansions of their *Or* repertoires, in particular in *L. acervorum* compared with *H. sublaevis* (fig. 2*C*, table 2, and supplementary table S16, Supplementary Material online). *Gr* expansions were also moderate across host species, but hosts exhibited significantly more species-specific duplications of *Gr*s compared with their respective parasite for two of the three species pairs in our study: *H. sublaevis* and *L. acervorum*, and *T. ravouxi* and *T. unifasciatus* (table 2).

Hosts retained similar numbers of *Or*s and *Gr*s compared with the nonhost species (figs. 2*B* and 3*B*), and no difference in *Or* or *Gr* turnover rate was found between hosts and nonhosts (exact binomial tests for host gain or loss compared with nonhosts: all *P*_adj_>0.05).

### Convergent Relaxation of Selection and Loss of Specific Receptors across Three Origins of Parasitism

For both chemoreceptor families, we tested whether convergent loss occurred more often than expected by chance. Convergent loss was defined as the occurrence of chemoreceptor loss in all three slave-making ant species within an orthologous cluster. In total, 18 out of 307 orthologous odorant receptor clusters showed convergent loss in the three slave-making ant species (fig. 2*D*), which was more than expected by chance (exact binomial test: expected prob. = 0.0164; observed prob. [95% CI] = 0.0586 [0.0351–0.0912]; *n* = 307; *P*=$4.433\times 10^{-6}$; power=0.8834). In contrast, not a single orthologous cluster underwent convergent loss in the hosts. This is not surprising given that loss was so rare in hosts that the probability that all three hosts lost the same *Or* by chance was only $7.949\times 10^{-5}$.

Previous studies on social and solitary Hymenoptera identified 30 *Or* subfamilies (fig. 2*A1*), each one representing one or a few genes that are ancestral to the ants, bees, and wasps (Zhou et al. 2012, 2015). We find that four of these ancestral clusters (red stars in fig. 2*A1*), were enriched for convergent *Or* loss in slave-making ants (table 3). These included the 9-exon subtree, which is the largest subtree in ants and is often implicated in the evolution of eusociality. Like the 9-exon *Or*s, the other three subfamilies enriched for convergent *Or* loss, that is, subtrees H, L, and P (fig. 2), have also been subject to gene family expansions in the ants (Engsontia et al. 2015; Zhou et al. 2015; Saad et al. 2018).

**Table 3.** Convergent Loss per Ancestral *Or* Subfamily.

(Sub-)family	Obs. Prob.	95% CI	*n*	*P*_adj_
All *Or*s	0.059	0.035–0.091	307	$4.433\times 10^{-6}$
9 exon	0.080	0.037–0.147	112	$9.697\times 10^{-4}$
H	0.250	0.032–0.651	8	0.0119
L	0.105	0.029–0.248	38	0.0119
P	0.286	0.037–0.710	7	0.0119

Note.—Statistics represent exact binomial test results. Only significant ancestral *Or*s are shown. Obs. prob., observed probability.

Thirteen out of 72 orthologous gustatory receptor clusters showed convergent loss in the three slave-making ant species (fig. 3*D*), which was expected by chance (exact binomial test: expected prob. = 0.1457; observed prob. [95% CI] = 0.1806 [0.0998–0.2889]; *n* = 72; *P* = 0.4027). No orthologous cluster underwent convergent loss in the hosts. To allow for comparisons between the two chemoreceptor families despite large differences in the total number of receptors, a power analysis was performed. Assuming a sample size of 307 orthologous clusters for *Gr*s, as is the case for *Or*s in our study, the probability of detecting convergent losses of specific *Gr*s above what is expected by chance was only 35% (i.e., power = 0.35 assuming *n* = 307 orthologous *Gr* clusters). In contrast, the probability of detecting significant convergent losses of specific *Or*s was 88%. This result highlights that the absence of detection of convergent loss for *Gr*s, but not for *Or*s, is not simply a statistical artifact of the lower sample size for *Gr*s. Rather, it reflects a fundamental difference in effect size (level of convergence) for *Gr*s and *Or*s.

**Fig. 4.**
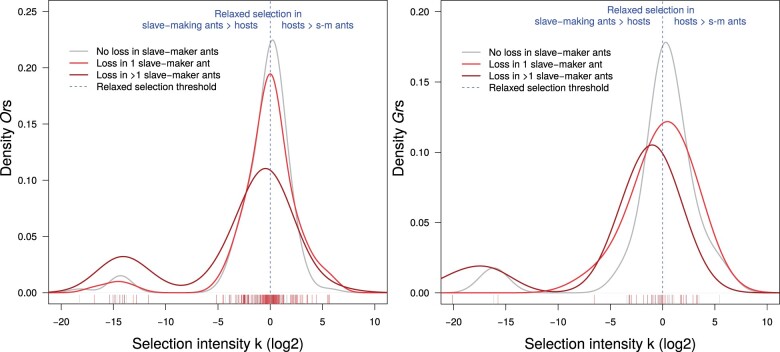
Intensity of selection acting on orthologous clusters with and without loss of slave-making ant *Or*s (left) and *Gr*s (right). The rug plot below each graph shows the distribution of the data points, and curves represent a smoothened density distribution. The intensity of selection on remaining slave-making ant orthologs decreased as more slave-making ant species lost an ortholog, that is, left-shift in the distribution from “No loss” to “Loss in >1 slave-making ant” (Wilcoxon rank sum test: *Gr*s: *W* = 226, *P* = 0.0138; *Or*s: *W* = 3,339.5, *P* = 0.0052). No significant difference was observed between clusters without loss and with loss in one slave-making species (*Gr*s: *W* = 133, *P* = 0.8672; *Or*s: *W* = 5,213, *P* = 0.8225).

Convergent relaxation of selection was extremely rare for both receptor families, with only three out of the 307 *Or* clusters and two out of the 72 *Gr* clusters showing evidence of convergent relaxation of selection in slave-making ants compared with hosts (all *k* < 1 and FDR < 0.05). However, comparing orthologous clusters where more than one slave-making ant had lost orthologs to those where no loss was observed revealed a decrease in the intensity of selection operating on the remaining slave-making ant orthologs (fig. 4; Wilcoxon rank sum test: *Gr*s: *W* = 226, *P* = 0.0136; *Or*s: *W* = 3,339.5, *P* = 0.0051). These results may suggest that convergent loss of slave-making ant *Or*s and *Gr*s is preceded by a gradual decay of nonessential receptor genes.

## Discussion

Chemoreceptors are pivotal to ant sociality, a key trait that facilitated the world-wide success of ants over the past approximately 100 My (Hölldobler and Wilson 1990; Ward 2014). Given their importance for mediating social communication and collaboration between colony members, the rapid expansion of chemoreceptors, and in particular the *Or*s, is thought to have facilitated the evolutionary transition from a solitary lifestyle to the building of complex societies in several social hymenopteran lineages (Smith, Zimin, et al. 2011; Smith CR, Smith CD, et al. 2011; Zhou et al. 2012; Engsontia et al. 2015; McKenzie et al. 2016; Pask et al. 2017; Trible et al. 2017; Yan et al. 2017, 2020). Our study demonstrates high turnover rates of chemoreceptors in all eight ant genomes, with up to 30% of orthologous *Gr*s undergoing expansions in hosts, and extensive losses of both *Or*s and *Gr*s in all three slave-making ants. This high turnover may facilitate rapid evolution as it provides variability in chemoreceptor repertoire on which selection can act. The dramatic losses of chemoreceptor genes we found in the parasitic slave-making ants show that the ongoing expansions of gene families involved in chemical communication is not merely halted, but actually reversed, in conjunction with the loss of important social traits.

We find that over 25% of the extensive *Or* repertoire was lost in each of the three independently evolved lineages of slave-making ants studied here. Losses occurred in 69% of all ancestral ant *Or*s and in 55% of the 307 *Or* orthologous clusters. These losses may reflect a diminished need to communicate during foraging and brood care tasks once slave-making ants started to rely on their host’s work force, and suggest that conserved genes that played an important role in the ancestor of the slave-making ant species studied here suddenly lost their function or even became maladaptive. Similarly high *Or* losses were recently reported for an inquiline socially parasitic ant, *Pseudoatta argentina* (Schrader et al. 2021), mirroring the genomic reduction frequently observed in parasites (Keeling and Slamovits 2005; International Helminth Genomes Consortium 2018). Thus, the transition to a parasitic lifestyle is associated with a loss of genes coding for traits that reflect the parasites’ dependence on their host, not only in well studied microparasites, but also in macroparasites like the ant social parasites.

We do not merely find dramatic shifts in *Or* repertoires in response to a change in the social environment, but these changes occurred repeatedly in the exact same *Or* orthologs along independent evolutionary lineages. Kapheim et al. (2015) studied the genomic underpinnings of social evolution in bees, where in contrast to the ants, eusociality evolved multiple times independently. They concluded that independent evolutionary transitions to a different social organization have independent genetic underpinnings (Kapheim et al. 2015). Similarly, when comparing the transcriptomes of three closely related slave-making ant species, Feldmeyer et al. (2017) found that distinct sets of genes were subject to positive selection in each slave-making ant lineage, underscoring the absence of genome-wide convergent trajectories during the evolution of the slave-making in ants. These findings stand in contrast to the convergent losses found along the three independent origins of social parasitism in our study, which demonstrate that the transition to a different social organization across independent lineages can go hand in hand with parallel genomic changes at the level of individual genes.

The fact that our species set represents three very recent and independent transitions to a different social organization allowed us to zoom in, not just to the level of >100-My-old ancestral families (Zhou et al. 2012, 2015; Engsontia et al. 2015; McKenzie et al. 2016; Branstetter et al. 2017), but on loss at the level of much more recent orthologous clusters. Nonetheless, investigations of these ancestral ant orthologs provide some interesting insights into the potential function of convergently lost *Or*s. Several previous studies show that expansion of chemoreceptors during social evolution is most prominent in the 9-exon *Or* subfamily, which is ancestral to all ants and plays an important role in social communication (Smith, Zimin, et al. 2011; Smith CR, Smith CD, et al. 2011; Engsontia et al. 2015; Zhou et al. 2015; McKenzie et al. 2016; Pask et al. 2017). This 9-exon subfamily was significantly enriched for convergent losses in the three slave-making ant species in our study. Other ancestral *Or* subfamilies implicated in the social organization of both ant colonies and bee hives are the H and P subfamilies (Zhou et al. 2015), which were similarly enriched for convergent loss in our slave-making ants (supplementary fig. S5*a*, Supplementary Material online).

*Gr*s are instrumental for sensing nutrients (Freeman et al. 2014; Xu 2019) and can be lost upon diet specialization. For example, losses of *Gr*s and, to a much lesser extent, of *Or*s have been reported in two fly species which specialized on certain food resources relative to their generalist sister species (McBride and Arguello 2007). Similarly, bees have relatively small *Gr* repertoires, potentially reflecting diminished need for gustatory perception due to mutualistic relationships with plants (Zhou et al. 2015). In ants, *Gr*s are primarily involved in the detection of soluble tastants, and are thus thought to play an important role during foraging and harvesting (Zhou et al. 2012). Because slave-making ants outsource these tasks to host workers, we hypothesized a decrease in *Gr* repertoire in slave-making ants. Nonetheless, the extent of *Gr* losses found in our slave-making ant species in recent evolutionary history is staggering: not only did slave-making ants lose about half of their *Gr* repertoire, losses occurred in no less then 76% of the orthologous *Gr* clusters and 64% of all *Gr*s that are ancestral to ants. Moreover, *Gr*s that were lost in some of our slave-making ant species were characterized by a decrease in the intensity of selection, indicating that *Gr* loss is an ongoing process and that slave-making ants are at different stages along the trajectory from gradual *Gr* decay under relaxation of selection, to the subsequent complete loss of *Gr*s. Similar high rates of *Gr* losses were recently reported for an inquiline social parasite (Schrader et al. 2021), which suggests that the transition to a socially parasitic lifestyle is accompanied by the loss of many nonessential *Gr*s.

Although the predominant mode of change in slave-making ant chemoreceptor repertoires was loss, only three *Or*s and two *Gr*s showed evidence for convergent relaxation of selection along all three parasitic lineages. Similarly, the intensity of selection was reduced for many chemoreceptor genes in slave-making ants, yet the extent to which chemoreceptors were released from selection compared with their host was lower than what was found recently in three inquiline socially parasitic ants (Schrader et al. 2021). This is remarkable because Schrader et al. (2021) focused on the typically more conserved single-copy orthologs, whereas we looked specifically at the highly dynamic chemoreceptor families. In fact, many of the *Or*s and *Gr*s found in our ants are subject to relaxed selection in hosts compared with slave-making ants (illustrated on the right-hand side of the graphs in fig. 4). In other words, these chemoreceptors are under stronger selection in slave-making ants compared with hosts. This suggests that some receptors underlie important functions specific to a parasitic lifestyle, such as host species recognition or social communication during slave raids (Regnier and Wilson 1971). These results underline that the chemoreceptor changes during the evolutionary transition to parasitism in slave-making ants is not governed exclusively by a release from natural selection, but potentially also by the retention of specific receptors which are instrumental to their success.

The question remains why convergent losses of *Or*s are more prevalent in slave-making ants than expected by chance, whereas convergent losses of *Gr*s are not. Our power analyses ruled out that this is merely an artifact of the smaller number of *Gr*s compared with *Or*s (72 and 307 orthologous clusters, respectively). Instead, the difference between both chemoreceptor families may reflect a distinction in the fundamental processes that govern their evolutionary dynamics in slave-making ants. One explanation could be that, unlike the *Gr*s, most *Or*s still serve a function and are retained in slave-making ants. This could result in an accumulation of losses in only those *Or*s that were released from selective pressure upon transitioning to a parasitic lifestyle. Nonetheless, this does not explain why the loss of *Or*s in two out of the three slave-making species did not differ from the null expectation that losses were randomly distributed over the *Or* phylogeny (*P* = 0.626), even though some of the remaining orthologs were subject to a decrease in selection intensity and thus presumably on the evolutionary trajectory to being lost.

Alternatively, the accumulation of convergent loss of *Or*s in slave-making ants may signify that some *Or*s did not merely lose their function, but that they actually impose a fitness disadvantage on their bearers. Indeed, *Or*s involved in nestmate and brood recognition could lead to the rejection of host brood and workers on which slave-making ant colonies so heavily depend. Similarly, *Or*s involved in queen pheromonal repression or worker policing to prevent workers from laying eggs may hamper worker reproduction so frequently seen in slave-making ants. Of course, we cannot reconstruct the evolutionary dynamics that preceded convergent loss in slave-making ant *Or*s, as any evidence was lost alongside these genes. However, our finding that ancestral ant *Or*s involved in social communication between colony members (Smith, Zimin, et al. 2011; Smith CR, Smith CD, et al. 2011; Zhou et al. 2012, 2015; Engsontia et al. 2015; McKenzie et al. 2016; Pask et al. 2017) are particularly enriched for convergent *Or* losses underscores that rapid, convergent losses of specific *Or*s could represent an adaptive response to a parasitic lifestyle.

The inference of absent genomic states (genes) is notoriously difficult, because absence of evidence is not the same as evidence of absence. We however minimized the risk of false negatives by obtaining high-quality PacBio-based assemblies, and by leveraging the benefits of sequence homology, synteny, gene expression, and manual annotation in a set of closely related species, using an iterative approach. This enabled us to recover between 349 and 546 chemoreceptors per species, which are among the largest chemoreceptor repertoires ever found in ants (McKenzie and Kronauer 2018). Indeed these repertoires are on par with those recently found in the highly eusocial leaf-cutting ants (Schrader et al. 2021), even though our ant species build relatively small societies with only a few dozen members and lack morphological variation in the worker caste. Given that our assembly and annotation methods were identical across all eight novel genomes and that the resulting genome assemblies are of similarly high quality for slave-making ants, hosts, and nonhosts, we are confident that the loss of 25–50% of the receptor repertoire in slave-making ants reliably reflects substantial and ongoing genomic reduction in chemoreceptors alongside the transition to a parasitic lifestyle.

In conclusion, we show that the evolutionary transition to a parasitic lifestyle went hand in hand with the convergent loss of a substantial part of the chemoreceptor repertoire along three independent origins of slave-making in ants. The loss of important social traits when adopting a parasitic lifestyle is reflected in the regression of the exact same ancestral genes that are often implicated in sociality. Collectively, these results demonstrate that evolution can act at the level of individual genes in a way that is both repeatable and reversible.

## Materials and Methods

### Sample Collection

To generate genomic data across three independent origins of slave-making in ants, colonies of three slave-making ant species (*H. sublaevis*, *T. ravouxi*, *T. americanus*), three host species (*L. acervorum*, *T. unifasciatus*, *T. longispinosus*), and two nonhost sister species (*T. rugatulus*, *T. nylanderi*) were collected in the United States and Central Europe (supplementary table S1, Supplementary Material online). Multiple samples were pooled for each species to meet the requirements for whole-genome sequencing. For each species, two samples were prepared for WGS: 1) Pacbio Sequel long read library and 2) Illumina paired-end library. Selected host colonies were unparasitized, free-living colonies consisting of only a single species: the focal host. In the parasite colonies, all brood should in principle be parasite brood because host workers do not produce offspring. Nonetheless, to rule out that any of the samples taken from parasite colonies are actually host brood (e.g., relics from previous so-called “slave raids”), only pupae of castes that are morphologically distinct from their host were selected for sequencing (i.e., queen pupae for *T. ravouxi*, and queen and worker pupae for *H. sublaevis* and *T. americanus*). Depending on the availability of pupae for each species and the need to pool DNA extractions due to low DNA content, between 28 and 156 pupal samples were obtained for WGS.

### Genome Sequencing

For each species, two samples were prepared for WGS: 1) PacBio Sequel long read library, aiming at 20 kb library construction and sequencing at 30× coverage; and 2) Illumina paired-end library with 150 bp long reads and 350 bp insert sizes. Only light colored, unscleratized pupae were selected for sequencing because 1) the lack of a hard cuticle may reduce the risk of shearing high molecular weight DNA and 2) pupae shed most of their gut content which reduces contamination by gut bacteria. Pupae were sampled from their colony as they developed (*∼*2-day intervals), snap frozen in liquid nitrogen, and stored at −80 °C. DNA extractions, quality checks, library preparation, and sequencing were performed by Novogene under the umbrella of the Global Ant Genome Alliance (Boomsma et al. 2017). For read statistics, see supplementary table S2, Supplementary Material online.

### Genome Assemblies and Polishing

Several genome assembly strategies were explored and compared based on assembly size, contiguity, and completeness (supplementary tables S3–S10, Supplementary Material online). For the final assembly, raw PacBio reads were assembled using the Canu (Koren et al. 2017) pipeline (parameter settings: correctedErrorRate = 0.15), with K-mer based genome size estimates. The Canu assemblies was polished with Pilon (version 1.22; parameter settings: diploid, fix = all; Walker et al. 2014), using Bowtie2-aligned Illumina short reads (version 2.3.4.1; Langmead and Salzberg 2012). Raw PacBio reads were mapped against the assemblies with Minimap2 (version 2.1; settings: -ax map-pb; Li 2018). Assemblies were then processed with Purge Haplotigs (Roach et al. 2018) and FinisherSC (in “fast” and “large” mode; Lam et al. 2015), followed by a final round of polishing with Pilon, Arrow (VariantCaller version 2.1.0), and again Pilon. Assembly contiguity and completeness were assessed with QUAST (version 3.1; Gurevich et al. 2013) and BUSCO (version 3.0.2; Simão et al. 2015), respectively.

### Genome Size Estimates

Genome sizes were estimated using the following two strategies: 1) The K-mer distribution of the Illumina libraries, for which we used the KmerCountExact utility of BBMap (Bushnell 2015). This analysis was run for K-mer sizes ranging from 31 to 131, selecting the largest genome size estimate as input for the Canu (Koren et al. 2017) genome assemblies. 2) The coverage of the PacBio-based assembly, where we mapped the original PacBio reads back to the genome assembly using Minimap2 (version 2.1; settings: -ax map-pb; Li 2018) and determined the coverage frequency distribution with the readhist module of Purge Haplotigs (Roach et al. 2018). The latter method is likely to yield higher and more correct genome size estimates than the former because large repetitive sequences are collapsed in the K-mer based estimate when they exceed Illumina read length. The coverage based genome size estimates of the final assemblies are very similar to the average genome size of Myrmicinae, which is 329.1 Mb (Tsutsui et al. 2008).

### Chemosensory Receptor Gene Annotation

Reference protein predictions were obtained from the eight species available on the Ant Genomes Portal: *Acromyrmex echinatior*, *Atta cephalotes*, *Camponotus floridanus*, *Cardiocondyla obscurior*, *Harpegnathos saltator*, *Linepithema humile*, *Pogonomyrmex barbatus*, and *Solenopsis invicta* (Elsik et al. 2015; supplementary table S11, Supplementary Material online). Reference protein predictions were functionally annotated based on their Pfam A domain content (i.e., gustatory receptors: “Trehalose_recp” and “7tm_7”; odorant receptors: “7tm_6”; Finn et al. 2015; version 31). Chemoreceptor genes were manually annotated using a two-pass tblastn/Exonerate—GeMoMa—WebApollo workflow. In the first pass, these reference protein predictions were blasted against the assemblies using tblastn (version 2.6.0; e-value = $1\times 10^{-3}$; Altschul et al. 1990). Annotations were then obtained with Exonerate (version 2.2.0; parameter settings: –model protein2genome; Slater and Birney 2005), which was run on those genomic regions with a blast hit. In parallel, we obtained GeMoMa (version 1.4.2; Keilwagen et al. 2016) annotations, based on reference protein predictions as well as the RNA-seq libraries for intron predictions. GeMoMa annotations were filtered using GAF, either retaining only complete gene models or all predictions (i.e., parameter settings: -r 0 -e 0). Annotations were filtered based on their Pfam A annotation, retaining only those genes that had the defining domains. The Exonerate, GeMoMa (i.e., all predictions), and GAF gene models, as well as the mapped RNA-seq reads were used as evidence tracks for manual annotation using WebApollo (version 2.1.0; Lee et al. 2013), which involved manually leveraging all homology and expression evidence to construct the best gene model for each prediction. In the second pass, the above workflow was repeated but now with all manual annotations from pass 1 as queries (i.e., from all focal species combined). We verified completeness of our chemoreceptor annotations by also constructing HMM profiles for insect *Gr*s and *Or*s based on sequences from the eight reference species mentioned above, and using these HMM profiles as query to scan our target genomes using HMMER (version 3.3, Eddy 2011). However, the HMM approach systematically returned fewer predictions than the manual annotation approach (supplementary tables S12 and S13, Supplementary Material online). Therefore, the set of manual annotations was used for all further analyses.

### Orthology Clustering

To identify orthologous clusters, we took an explicit phylogenetic approach using the Python module ETE3 (version 3.1.1; Huerta-Cepas et al. 2016). Specifically, multiple sequence alignments of odorant receptor (Or) and gustatory receptor (Gr) protein sequences from the eight focal species and nine reference species were obtained with MAFFT (version 7.310; Katoh and Standley 2013; parameter settings: –maxiterate 1,000 –localpair). Gene trees were constructed with Fasttree (version 2.1; Price et al. 2010; parameter setting: –pseudo) and IQTree (version 1.6.1 with the best-fit model automatically selected by ModelFinder; Minh et al. 2020), and rooted with the Or co-receptor and the Trehalose receptors, for the Or and Gr tree respectively. The FastTree trees were traversed using ETE3 and a clade was labeled as an orthologous cluster if a receptor from a reference species was found as outgroup. In total, 74% of the 379 orthologous clusters were 100% correctly identified and only 2.1% were completely missed. All clusters were thereafter manually curated and split or merged where necessary (for further details, see supplementary section 3, Supplementary Material online). Next, lowly supported branches (bootstrap value <0.7) and species-specific expansions were collapsed. FastTree-based orthologous group predictions were cross-referenced with those based on IQTree. This yielded 100% identical orthologous groups for >99% of the Or orthologous groups and 98.8% of the Gr orthologous groups, demonstrating that the orthology group definitions were robust. Excluding the orthologous groups that differed between FastTree and IQTree (not shown) did not qualitatively change the results of downstream analyses.

### Identification of Losses and Gains

Two parallel approaches were used for the identification of receptor gene gains and losses: 1) using tree reconciliation (ETE3 version 3.1.1; Huerta-Cepas et al. 2016); and 2) based on phylogenetically identified orthologous clusters (methods detailed below). The results of these two approaches were qualitatively identical, although tree reconciliation resulted in a much higher number of inferred gene losses, especially within the closely related *Temnothorax* clade (supplementary tables S14 and S15, Supplementary Material online). These results demonstrate there is a limit to the phylogenetic signal present in these large and complex gene families shared among closely related species. In the main text, we therefore present the more conservative estimates based on orthologous clusters, for which the phylogenetic signal was strong.

For each orthologous cluster, we first identified and collapsed species-specific expansions. If branches were collapsed because of low (<0.7) bootstrap support (i.e., tri-/multifurcations), then in-paralogs or nested in-paralogs were also identified and collapsed. Any orthologous cluster for which in-paralogs were identified and collapsed in any host or parasite species was defined as an orthologous cluster where gain had occurred for that species. Next, we computed the difference between the number of remaining (noncollapsed) slave-making ant paralogs and those found in the respective host. For example, if an orthologous group contained one ortholog in the host and 0 orthologs in the respective slave-making ant, the difference is <0, indicating loss has occurred in this parasite for this orthologous cluster. For partial out-paralogs (i.e., those assigned to the same orthologous cluster because the duplication event occurred in the common ancestor of some, but not all of our focal species), this translates into a parasite having lost one or more out-paralogs compared with its host. Thus, for each orthologous cluster, parasite loss is defined as the No. parasite orthologs – No. corresponding host orthologs <0, and host loss as No. parasite orthologs – No. corresponding host orthologs >0 (see also supplementary fig. S4, Supplementary Material online). This way, for each orthologous cluster, and for each of the slave-making ant species and host species, we defined whether or not gain and/or loss had occurred.

### Test of Convergent Losses and Gains

For both chemosensory receptor families, we tested whether convergent loss occurred more often then expected by chance. The probability of loss for each species was defined as the proportion of orthologous clusters where loss occurred in that species. The expected probability of convergent loss is the probability that loss occurred along all three origins of slavery, independently, so just by chance. This was defined as the product of the probabilities of loss in all three slave-making ants or all three hosts. The observed probability then is the fraction of the orthologous groups where loss was actually observed simultaneously in all three slave-making ant species or all three hosts. If the observed probability is significantly higher than expected based on a binom.test (R version 3.6.2; R Core Team 2020), this is taken as evidence for convergent loss in the *Or* or *Gr* receptor family.

### Relaxed Selection Analyses

Relaxation of natural selection was assessed for each of the 307 *Or* orthologous clusters and 72 *Gr* orthologous clusters. Protein alignments were obtained with Prank (version v.170427; Löytynoja 2013) and nucleotide alignments with Pal2Nal (version 14; Suyama et al. 2006). Approximate maximum likelihood phylogenies were generated with FastTree and used as guide tree in the relaxed selection analyses using HyPhy RELAX (version 2.5.31; Wertheim et al. 2014). RELAX assesses whether the d*N*/d*S* of test branches (slave-making ants) differs from the d*N*/d*S* of reference branches (hosts) and infers a selection intensity parameter *k*. A *k* < 1 and FDR < 0.05 mean that slave-making ant branches were subject to relaxation of selection relative to host branches, whereas a *k* > 1 and FDR < 0.05 mean that slave-making ant branches were subject to intensification of selection relative to host branches. Orthologous groups with at least one d*N*/d*S* > 10 were excluded from the analyses.

## Supplementary Material

Supplementary data are available at *Molecular Biology and Evolution* online.

## Supplementary Material

msab305_Supplementary_Data
