# Supplementary material for: Convergent Loss of Chemoreceptors across Independent Origins of Slave-Making in Ants
Source: Mol Biol Evol. 2021 Oct 20;39(1):msab305. doi: 10.1093/molbev/msab305 (PMC8760941; doi:10.1093/molbev/msab305)
Supplement: msab305_Supplementary_Data [file msab305_supplementary_data.zip › jongepier_bornbergbauer_slavery_receptor_loss_SUPPL_20210831_revision.pdf]

Convergent loss of chemoreceptors across independent origins of slave-making in ants

SUPPLEMENTARY MATERIAL

Evelien Jongepier[a,b,*], Alice Séguret[a], Anton Labutin[a], Barbara Feldmeyer[c], Claudia Gstöttl[d], Susanne Foitzik[e], Jürgen Heinze[d], and Erich Bornberg-Bauer[a]

[a]Institute for Evolution and Biodiversity, Westfälische Wilhelms University, Münster, Germany

[b]Institute for Biodiversity and Ecosystem Dynamics, University of Amsterdam, Amsterdam, The Netherlands

[c]Senckenberg Biodiversity and Climate Research Centre, Frankfurt am Main, Germany

[d]Institute for Zoology, University of Regensburg, Regensburg, Germany

[e]Institute of Organismic and Molecular Evolution, Johannes Gutenberg University, Mainz, Germany

[*]Corresponding author: e.jongepier@uva.nl

1 Genome assemblies

Several genome assembly strategies were explored and compared based on assembly size (section Genome size estimates), contiguity (assessed with `QUAST`; version 3.1; Gurevich et al., 2013) and completeness (assessed with `BUSCO`; version 3.0.2; insect db; Simão et al., 2015). Of particular concern was that several assembly sizes were (much) larger than expected (see section Genome size estimates) and these assemblies contained a high number of duplicated orthologs which usually occur as single-copy genes in insect genomes (*i.e.* duplicated `BUSCO`s). This assembly inflation is likely due to the fact that several individuals had to be pooled in order to obtain enough high molecular weight DNA for these tiny ants (section Sample collection). Thus, the samples used for PacBio library prep were polyploid, which can result in the same polymorphic genomic region being represented in the assembly as separate contigs, rather than as a contig and an associated haplotig. The main aim of the following assembly strategies was thus to reduce redundancy and obtain a haploid genome assembly for each of our focal species.

1. `MaSuRCA`: For most of our focal species, a preliminary assembly was generated with `MaSuRCA` (version 3.2.6;

default parameter settings; Zimin et al., 2013) based on the raw, untrimmed Illumina reads only. This assembly served to error correct the PacBio reads (see 2).

2. `HALC-CANU`: Raw PacBio reads were error corrected prior to assembly with the help of the preliminary `MaSuRCA` assembly. The PacBio reads were then aligned to the `MaSuRCA` assembly using `Blasr` (`smrtanalyses` version 2.3.0; parameter settings: maxScore=2000, minMatch=8, nCandidates=30, bestn=20, m=5; Chaisson and Tesler, 2012). Aligned reads were error-corrected with `HALC` (date: 04.2018; default parameter settings of the `HALC` wrapper script; Bao and Lan, 2017), making use of LorDEC (version 0.7; Salmela and Rivals, 2014). The resulting corrected, trimmed and split PacBio reads were then assembled using `CANU` (version 1.7; Koren et al., 2017; default parameter settings and the K-mer based genome size estimates, see section Genome size estimates).
3. $\texttt{CANU}_{\texttt{default}}$: Raw PacBio reads were error corrected, trimmed, split and assembled using the `CANU` [Koren et al., 2017] pipeline with default parameter settings and the K-mer based genome size estimates (see section Genome size estimates).
4. $\texttt{CANU}_{\texttt{high error}}$: Raw PacBio reads were error corrected, trimmed, split and assembled using the `CANU` [Koren et al., 2017] pipeline (using K-mer based genome size estimates, see section Genome size estimates). Instead of using default parameter settings as in 2, we tweaked the `CANU` error rate and coverage settings in order to smash haplotypes together (`correctedErrorRate=0.15, corOutCoverage=200`; see also Canu FAQ (here)).

Not all assembly methods were applied to all species: assemblies were skipped if we had already decided to proceed with an alternative approach based on results in the other species (indicated by empty columns in Supplementary tables S3-S10).

2 Genome polishing

The `Canu` assemblies were polished with `Pilon` (version 1.22; parameter settings: `diploid, fix=all`; Walker et al., 2014), using the Illumina short reads, which were first aligned to the `Canu` assembly using `Bowtie2` (version 2.3.4.1; Langmead and Salzberg, 2012).

The $\texttt{CANU}_{\texttt{high error}}$ assemblies was further processed with `Purge Haplotigs` (Roach et al., 2018) to identify syntenic pairs of contigs and discard one of them. Hereto, the raw PacBio reads were mapped against the `Canu` genome assembly with `Minimap2` (version 2.1; settings: `-ax map-pb`; Li, 2018). Assessment of genome read coverage and read depth cutoffs, as well as the actual purging of haplotigs was done with the `Purge Haplotigs` pipeline [Roach et al., 2018].

Coverage frequency distributions provide insight into ploidy: In a haploid genome assembly where each genomic location is represented only once, the coverage frequency distribution is unimodal and symmetrical around its mode.

If the same genomic location is erroneously assembled twice due to heterozygosity in diploid samples, then each of these contigs will only get half of the coverage, causing a second mode at half the coverage of the haploid peak. Given that we had polyploid rather than diploid source material, no bimodality in the read depth frequency distributions could be observed. Instead, an over-representation of low coverages was observed in the $\texttt{CANU}_{\texttt{default}}$, and to a lesser extent in $\texttt{CANU}_{\texttt{high error}}$, but not in the final `Purge Haplotigs` polished assemblies. Importantly, purging haplotigs did not affect `BUSCO` scores, with the exception of a substantial drop in duplicated `BUSCO`s (Tables S3-S10).

The `Purge Haplotigs`-polished assemblies were then upgraded with `FinisherSC` (in "fast" and "large" mode; Lam et al., 2015), followed by a final round of polishing with `Arrow` (`VariantCaller` version 2.1.0) and `Pilon` (version 1.22; parameter settings: `diploid, fix=all`; Walker et al., 2014). `Arrow` can correct large structural errors as it is based on the raw PacBio reads. `Quiver` aims to correct small errors based on the short, but high quality Illumina reads. To run `Arrow`, raw PacBio reads were aligned to the assembly with `Minimap2` and converted to a PacBio-compatible bam using `pbbamify` (version 0.13.2).

3 Orthology and Phylogeny

Preliminary analyses using `OrthoMCL` or `OrthoFinder` yielded very poor orthology clustering results. This is probably due to the large number of paralogs, and the close relatedness among our focal species. Visual inspection of the Or and Gr protein trees however showed that clear orthologous clusters could be identified, especially when the receptors of more distantly related outgroup species were considered. We therefore chose to use an explicit phylogenetic approach to identify orthologous clusters using the `Python` module `ETE3` (version 3.1.1; Huerta-Cepas et al. 2016). Specifically, multiple sequence alignments of Or and Gr protein sequences from the eight focal species and nine reference species (using reference genomes available on the Ant Genomes Portal) were obtained with `MAFFT` (version 7.310; Katoh and Standley 2013; parameter settings: `--maxiterate 1000 --localpair`). Gene trees were constructed with `Fasttree` (version 2.1; Price et al. 2010; parameter setting: `--pseudo`) and rooted based on the Or co-receptor and the Trehalose receptors, for the *Or* and *Gr* tree respectively. The trees were traversed using `ETE3` and a clade was labelled as an orthologous cluster if a receptor from a reference species was found as outgroup.

Obtaining a single multiple sequence alignment for all 4 759 *Or*s present in the nine reference and eight focal species required more than the available 756 GB of RAM. Therefore, (i) the Or protein tree of the eight focal species was split into four sub-trees. (ii) The Or protein sequences of the reference species were `BLASTP`-ed against the Or protein sequences of the focal species. (iii) Each reference species Or was assigned to the same sub-tree as its best blast hit, yielding four separate sets of Or protein sequences from all 17 species. Multiple sequence alignment, phylogenetic tree construction and orthology assignment were then done as described above for each of the four sub-trees separately.

Visual inspection confirmed correct automated identification of 58.3% the 72 final orthologous Gr clusters and 77.5% of the 307 orthologous Or clusters. Manual curation resulted in the merging of five Gr clusters, the identification of an additional four clusters, and the splitting of eleven initial clusters in two or more orthologous groups based on the presence of *H. sublaevis* and/or *L. acervorum* as an outgroup to the other focal species. For the Ors, manual curation resulted in 25 mergings, six splits and the identification of four additional orthologous clusters.

Table S1: Sample information.

Species	Colony ID	Latitude	Longitude	Collection date	Sample
Harpagoxenus sublaevis	Hsub_1	49°20'40.4"	11°15'37.4"	10-05-2017	28 female pupae
Leptothorax acervorum	Lace_23	49°21'42.0"	11°17'17.0"	10-05-2017	75 pupae
Temnothorax ravouxi	Mrav_6	49°00'39.0"	11°57'12.0"	21-06-2018	156 queen pupae
Temnothorax unifasciatus	Tuni_6	49°09'51.2"	11°56'49.7"	06-10-2016	112 pupae
Temnothorax americanus	Tame_NY17_H226	42°31'36.9"	-74°10'15.6"	25-05-2017	42 female pupae
Temnothorax longispinosus	Tlon_NY17_J1612	42°31'36.9"	-74°10'15.6"	25-05-2017	136 pupae
Temnothorax rugatulus	Trug_M654	31°51'1.8"	-109°19'32.2"	20-08-2015	140 pupae
Temnothorax nylanderi	Tnyl_W156	49°48'38.0"	7°52'07.9"	15-08-2016	148 pupae

Table S2: Genomic read statistics.

	Illumina reads		PacBio reads			
species	Gbases	No. read pairs	Gbases	No. reads	N50	N90
Harpagoxenus sublaevis	18.076	60 254 049	16.094	2 758 201	8 940	3 423
Leptothorax acervorum	15.666	52 218 260	16.353	2 077 285	12 114	4 805
Temnothorax ravouxi	16.358	54 527 419	24.727	2 810 494	11 186	5 900
Temnothorax unifasciatus	16.636	55 454 527	14.647	2 115 430	10 295	4 256
Temnothorax americanus	11.725	38 825 160	13.919	1 721 559	10 692	5 280
Temnothorax longispinosus	28.691	95 002 424	19.476	2 579 317	10 916	4 465
Temnothorax rugatulus	15.664	52 214 755	18.610	2 282 653	11 733	4 900
Temnothorax nylanderi	18.267	60 889 461	17.148	2 002 476	12 477	5 111

Table S3: *Harpagoxenus sublaevis* assembly statistics. The column `MaSuRCA` contains the statistics for the assembly generated with `MaSuRCA`. The column `HALC-Canu`$_{def}$ contains the statistics for the assembly generated using `CANU` with default parameter settings, using reads error-corrected with `HALC`. The column `Canu`$_{def}$`-Pilon` contains statistics for the assembly generated using `CANU` with default parameter settings, followed by a round of polishing with `Pilon`. The column `Canu`$_{err}$`-Pilon` indicates statistics for the assembly generated using `CANU` with high error rates, followed by a round of polishing with `Pilon`. The `Canu`$_{err}$ assemblies were further processed with `Purge Haplotigs` (statistics presented in the column `PurgeHaplotigs`), then upgraded with `FinisherSC` (statistics presented in column `FinisherSC`) and a final round of polishing with `Arrow` and `Pilon` (statistics presented in the last column). Not all assembly methods were applied to all species (hence the empty columns) because this was not necessary to evaluate the performance of the applied methods. Only the assembly presented in the last column was used for further analyses.

Assembly	MaSuRCA	HALC-Canu$_{def}$	Canu$_{def}$-Pilon	Canu$_{err}$-Pilon	PurgeHaplotigs	FinisherSC	Arrow-Pilon
No. contigs	164 918	-	7 355	5 956	2 794	2 750	2 750
Total length	376 588 678	-	429 220 703	392 891 886	341 470 629	341 284 157	341 523 168
Largest contig	87 368	-	6 171 555	4 362 322	4 362 322	4 360 839	4 363 893
GC (%)	39.10	-	39.15	39.17	39.00	39.00	39.00
N50	5 179	-	224 287	260 672	390 745	390 729	391 101
N75	1 925	-	42 041	54 059	94 152	94 153	94 448
L50	18 273	-	327	258	176	176	176
L75	47 420	-	1 503	1 195	651	650	649
Complete BUSCO's (%)	87.8	-	98.1	98.2	98.1	97.5	98.1
Complete, single copy BUSCO's (%)	86.3	-	86.9	94.3	96.7	96.3	96.7
Complete, duplicated BUSCO's (%)	1.5	-	11.2	3.9	1.4	1.2	1.4
Fragmented BUSCO's (%)	8.5	-	0.7	0.7	0.7	1.2	0.7
Missing BUSCO's (%)	3.7	-	1.2	1.1	1.2	1.3	1.2

Table S4: *Leptothorax acervorum* assembly statistics. For further details, see Table S3 legend.

Assembly	MaSuRCA	HALC-Canu$_{def}$	Canu$_{def}$-Pilon	Canu$_{err}$-Pilon	PurgeHaplotigs	FinisherSC	Arrow-Pilon
No. contigs	79 589	10 676	7 544	5 433	1 545	1 269	1 269
Total length	348 003 143	411 018 547	516 250 221	455 819 920	346 747 927	345 140 810	344 708 930
Largest contig	382 012	774 699	4 002 204	6 338 506	6 338 506	6 338 506	6 338 531
GC (%)	38.57	38.51	38.58	38.66	38.68	38.67	38.67
N50	21 005	67 835	211 559	298 336	605 042	729 587	730 108
N75	4 656	27 793	43 009	65 476	194 900	247 721	248 295
L50	3 332	1 301	458	247	119	104	104
L75	12 722	3 865	1 990	1 140	380	310	310
Complete BUSCO's (%)	95.3	-	98.2	98.3	98.1	98.0	98.3
Complete, single copy BUSCO's (%)	94.5	-	80.9	92.1	96.0	96.1	96.7
Complete, duplicated BUSCO's (%)	0.8	-	17.3	6.2	2.1	1.9	1.6
Fragmented BUSCO's (%)	2.8	-	0.8	0.7	0.7	0.7	0.6
Missing BUSCO's (%)	1.9	-	1.0	1.0	1.2	1.3	1.1

Table S5: *Temnothorax ravouxi* assembly statistics. For further details, see Table S3 legend.

Assembly	MaSuRCA	HALC-Canu$_{def}$	Canu$_{def}$-Pilon	Canu$_{err}$-Pilon	PurgeHaplotigs	FinisherSC	Arrow-Pilon
No contigs	-	-	-	18 188	5 018	4 380	4 380
Total length	-	-	-	596 230 198	366 250 529	362 215 514	362 408 415
Largest contig	-	-	-	1 215 712	1 216 519	1 285 007	1 282 434
GC (%)	-	-	-	38.67	38.69	38.69	38.70
N50	-	-	-	51 549	106 656	128 643	128 698
N75	-	-	-	22 875	56 975	66 149	66 202
L50	-	-	-	2 477	901	751	751
L75	-	-	-	7 007	2 087	1 733	1 733
Complete BUSCO's (%)	-	-	-	-	-	-	95.7
Complete, single copy BUSCO's (%)	-	-	-	-	-	-	88.0
Complete, duplicated BUSCO's (%)	-	-	-	-	-	-	7.7
Fragmented BUSCO's (%)	-	-	-	-	-	-	1.2
Missing BUSCO's (%)	-	-	-	-	-	-	3.1

Table S6: *Temnothorax unifasciatus* assembly statistics. For further details, see Table S3 legend.

Assembly	MaSuRCA	HALC-Canu$_{def}$	Canu$_{def}$-Pilon	Canu$_{err}$-Pilon	PurgeHaplotigs	FinisherSC	Arrow-Pilon
No. contigs	47 056	12 586	-	5 915	2 964	2 496	2 496
Total length	294 647 559	464 253 540	-	365 527 575	317 439 015	314 940 905	314 490 334
Largest contig	347 969	1 205 371	-	2 812 578	2 812 578	2 906 488	2 901 263
GC (%)	39.20	38.67	-	38.72	38.80	38.80	38.81
N50	25 189	66 013	-	214 554	290 891	360 251	359 731
N75	8 152	25 538	-	48 796	92 794	117 848	117 842
L50	2 862	1 359	-	332	236	196	196
L75	8 037	4 470	-	1 278	733	587	587
Complete BUSCO's (%)	94.1	-	-	98.4	98.0	98.0	98.2
Complete, single copy BUSCO's (%)	93.5	-	-	92.1	95.3	95.2	95.4
Complete, duplicated BUSCO's (%)	0.6	-	-	6.3	2.7	2.8	2.8
Fragmented BUSCO's (%)	3.9	-	-	0.5	0.8	0.8	0.6
Missing BUSCO's (%)	2.0	-	-	1.1	1.2	1.2	1.2

Table S7: *Temnothorax americanus* assembly statistics. For further details, see Table S3 legend.

Assembly	MaSuRCA	HALC-Canu$_{def}$	Canu$_{def}$-Pilon	Canu$_{err}$-Pilon	PurgeHaplotigs	FinisherSC	Arrow-Pilon
No. contigs	33 648	7 960	5 585	4 202	1 829	1 229	1 229
Total length	267 639 222	370 679 867	358 839 550	335 049 332	287 572 788	279 748 504	279 543 510
Largest contig	267 332	1 880 024	3 579 705	4 384 607	4 384 607	4 384 607	4 381 237
GC (%)	39.08	39.19	39.14	39.21	39.21	39.20	39.20
N50	28 826	122 674	235 295	369 006	509 480	603 326	603 026
N75	11 469	30 921	47 521	78 171	166 114	247 685	247 480
L50	2 514	579	321	199	146	121	121
L75	6 147	2 256	1 158	706	396	298	298
Complete BUSCO's (%)	94.9	-	98.1	98.2	97.7	97.8	98.2
Complete, single copy BUSCO's (%)	94.2	-	86.0	92.0	96.1	96.2	96.9
Complete, duplicated BUSCO's (%)	0.7	-	12.1	6.2	1.6	1.6	1.3
Fragmented BUSCO's (%)	3.6	-	0.7	0.7	0.8	0.9	0.6
Missing BUSCO's (%)	1.5	-	1.2	1.1	1.5	1.3	1.2

Table S8: *Temnothorax longispinosus* assembly statistics. For further details, see Table S3 legend.

Assembly	AllPaths-LG	HALC-Canu$_{def}$	Canu$_{def}$-Pilon	Canu$_{err}$-Pilon	PurgeHaplotigs$_{err}$	PurgeHaplotigs$_{def}$	FinisherSC	Arrow-Pilon
No contigs	22 624	-	8 013	7 938	6 402	4 012	3 430	3 430
Total length	238 236 400	-	363 746 299	275 125 770	257 965 379	300 694 620	298 389 766	298 344 416
Largest contig	284 717	-	1 040 803	490 944	490 944	1 040 803	1 040 803	1 041 114
GC (%)	38.78	-	38.84	39.13	39.14	38.94	38.94	38.93
N50	30 134	-	101 476	56 724	61 264	129 642	156 583	156 499
N75	11 243	-	35 819	29 170	33 104	64 750	77 995	78 015
L50	2 153	-	918	1 412	1 267	644	541	541
L75	5 361	-	2 426	3 099	2 693	1 456	1 211	1 212
Complete BUSCO's (%)	95.5	-	94.8	82.6	82.3	94.0	93.7	95.6
Complete, single copy BUSCO's (%)	94.9	-	89.0	82.0	81.9	92.7	92.4	93.8
Complete, duplicated BUSCO's (%)	0.6	-	5.1	0.6	0.4	1.3	1.3	1.8
Fragmented BUSCO's (%)	2.4	-	1.8	3.5	3.6	2.1	2.0	1.1
Missing BUSCO's (%)	2.1	-	3.4	13.9	14.1	3.9	4.3	3.3

Table S9: *Temnothorax rugatulus* assembly statistics. For further details, see Table S3 legend.

Assembly	MaSuRCA	HALC-Canu$_{def}$	Canu$_{def}$-Pilon	Canu$_{err}$-Pilon	PurgeHaplotigs	FinisherSC	Arrow-Pilon
No contigs	135 073	13 919	8 722	6 535	2 358	2 079	2 079
Total length	385 821 052	463 678 274	467 986 203	424 544 191	336 328 124	335 682 764	335 735 536
Largest contig	352 616	731 202	3 107 326	4 507 738	4 507 738	4 507 738	4 513 070
GC (%)	39.45	38.94	39.16	39.28	39.33	39.32	39.31
N50	7 244	53 509	135 322	235 761	425 938	450 151	450 769
N75	2 502	25 720	35 521	46 820	118 981	140 517	141 030
L50	12 870	1 874	584	314	170	155	155
L75	35 050	5 135	2 515	1 503	554	494	493
Complete BUSCO's (%)	90.0	-	98.1	98.5	98.4	98.3	98.4
Complete, single copy BUSCO's (%)	88.4	-	83.9	91.8	96.0	95.7	96.1
Complete, duplicated BUSCO's (%)	1.6	-	14.2	6.7	2.4	2.6	2.3
Fragmented BUSCO's (%)	7.3	-	0.6	0.6	0.6	0.6	0.7
Missing BUSCO's (%)	2.7	-	1.3	0.9	1.0	1.1	0.9

Table S10: *Temnothorax nylanderi* assembly statistics. For further details, see Table S3 legend.

Assembly	MaSuRCA	HALC-Canu$_{def}$	Canu$_{def}$-Pilon	Canu$_{err}$-Pilon	PurgeHaplotigs	FinisherSC	Arrow-Pilon
No. contigs	63 870	-	5 108	3 357	1 785	1 555	1 555
Total length	299 554 352	-	410 563 087	346 999 125	312 275 949	313 533 164	313 389 767
Largest contig	337 061	-	3 912 986	4 275 559	4 275 559	4 919 227	4 914 139
GC (%)	39.24	-	38.84	38.98	39.07	39.06	39.06
N50	21 722	-	265 791	390 170	542 134	618 788	619 324
N75	5 290	-	55 617	108 502	162 861	198 066	197 671
L50	3 035	-	289	161	123	111	111
L75	10 343	-	1 194	605	405	349	349
Complete BUSCO's (%)	94.8	-	98.4	98.3	98.3	98.2	98.5
Complete, single copy BUSCO's (%)	94.1	-	86.8	95.1	97.1	95.7	96.1
Complete, duplicated BUSCO's (%)	0.7	-	11.6	3.2	1.2	2.5	2.4
Fragmented BUSCO's (%)	3.1	-	0.5	0.6	0.7	0.7	0.6
Missing BUSCO's (%)	2.1	-	1.1	1.1	1.0	1.1	0.9

Table S11: Reference species set for which protein predictions were obtained from the Ants Genomes Portal [Elsik et al., 2015].

Species	Version	Reference
Acromyrmex echinatior	Official Gene Set v3.8	Nygaard et al. 2011
Atta cephalotes	Official Gene Set v1.2	Suen et al. 2011
Camponotus floridanus	Official Gene Set v3.3	Bonasio et al. 2010
Cardiocondyla obscurior	Official Gene Set v 1.4	Schrader et al. 2014
Harpegnathos saltator	Official Gene Set v3.3	Bonasio et al. 2010
Linepithema humile	Official Gene Set v1.2	Smith et al. 2011a
Pogonomyrmex barbatus	Official Gene Set v1.2	Smith et al. 2011b
Solenopsis invicta	Official Gene Set v2.2.3	Wurm et al. 2011

Table S12: Number of *Or* gene predictions in each species obtained with the HMM approach *versus* the manual annotation approach. Various thresholds were tested regarding the percentage of the predicted *Or* sequence required to overlap with the HMM, ranging from 90% of the predicted sequence down to a more relaxed threshold of 60%. In all cases, the amount of predictions recovered with the HMMER method was far lower than the amount of annotations obtained with the manual annotation method, shown in the last column.

	HMM coverage				
Species	90%	80%	70%	60%	Manual annotations
H. sublaevis	11	28	63	125	309
L. acervorum	14	41	80	144	403
T. ravouxi	9	24	59	118	315
T. unifasciatus	19	36	71	152	414
T. americanus	7	20	43	110	308
T. longispinosus	10	33	65	137	419
T. rugatulus	14	35	73	144	421
T. nylanderi	16	33	63	138	418

Table S13: Number of *Gr* gene predictions in each species obtained with the HMM approach *versus* the manual annotation approach. Various thresholds were tested regarding the percentage of the predicted *Gr* sequence required to overlap with the HMM, ranging from 90% of the predicted sequence down to a more relaxed threshold of 60%. In all cases, the amount of predictions recovered with the HMMER method was far lower than the amount of annotations obtained with the manual annotation method, shown in the last column.

	HMM coverage				
Species	90%	80%	70%	60%	Manual annotations
H. sublaevis	8	31	34	41	52
L. acervorum	17	75	81	91	127
T. ravouxi	7	28	30	32	49
T. unifasciatus	25	87	92	95	117
T. americanus	5	14	15	18	41
T. longispinosus	15	50	54	55	91
T. rugatulus	22	63	65	69	106
T. nylanderi	28	69	72	75	128

Table S14: Number of *Or*s observed in each of the focal species, and inferred based on tree reconciliation. Two phylogenetic tree construction algorithms were used: `IQTree` and `FastTree`, which yielded highly comparable results. The number of inferred losses is very high, in particular in the *Temnothorax clade.* Visual inspection confirmed that this is due to inconsistent topology between ortholog trees and species trees within this closely related species set.

		`FastTree`		`IQTree`	
Species	Observed No. *Or*s	in reconciliated tree	inferred losses (%)	in reconciliated tree	inferred losses (%)
H. sublaevis	309	484	175 (36.2)	503	194 (38.57)
L. acervorum	403	484	98 (19.6)	517	114 (22.05)
T. ravouxi	315	501	1037 (76.7)	1358	1043 (76.80)
T. unifasciatus	414	1352	910 (68.7)	1331	917 (68.9)
T. americanus	308	1303	1055 (77.4)	1368	1060 (77.5)
T. longispinosus	419	1363	934 (69.0)	1359	940 (69.2)
T. rugatulus	421	1324	894 (68.0)	1320	899 (68.1)
T. nylanderi	418	1315	885 (67.9)	1306	888 (68.0)
Total	3007	1353	5988(66.6)	9062	6055(66.8)

Table S15: Number of *Gr*s observed in each of the focal species, and inferred based on tree reconciliation. Two phylogenetic tree construction algorithms were used: `IQTree` and `FastTree`, which yielded highly comparable results. The number of inferred losses is very high, in particular in the *Temnothorax* clade. Visual inspection confirmed that this is due to inconsistent topology between ortholog trees and species trees within this closely related species set.

		`FastTree`		`IQTree`	
Species	Observed No. *Gr*s	in reconciliated tree	inferred losses (%)	in reconciliated tree	inferred losses (%)
H. sublaevis	52	130	78 (60.0)	136	84 (61.8)
L. acervorum	127	153	26 (17.0)	157	30 (19.1)
T. ravouxi	49	286	237 (82.9)	289	240 (83.0)
T. unifasciatus	117	299	182 (60.9)	303	186 (61.4)
T. americanus	41	285	244 (85.6)	288	247 (85.8)
T. longispinosus	91	290	199 (68.6)	291	200 (68.7)
T. rugatulus	106	285	179 (62.8)	288	182 (63.2)
T. nylanderi	128	287	159 (55.4)	292	164 (56.2)
Total	711	2015	1304(64.7)	2044	1333(65.2)

Table S16: Proportion of chemoreceptor orthologous clusters which exhibited gene losses or gains in each of the focal species. In slave-making ant species, the proportion was determined in relation to their respective host and outgroup species, and for hosts in relation to their respective slave-making ant and outgroup species.

Species	Proportion *Or*s lost (%)	Proportion *Or*s gained (%)	Proportion *Gr*s lost (%)	Proportion *Gr*s gained (%)
H. sublaevis	25.08	1.30	55.56	4.17
L. acervorum	3.26	7.49	0	29.17
T. ravouxi	27.69	6.19	47.22	1.14
T. unifasciatus	7.49	7.82	8.33	13.89
T. americanus	29.97	2.93	44.44	1.14
T. longispinosus	7.49	5.21	4.17	6.94

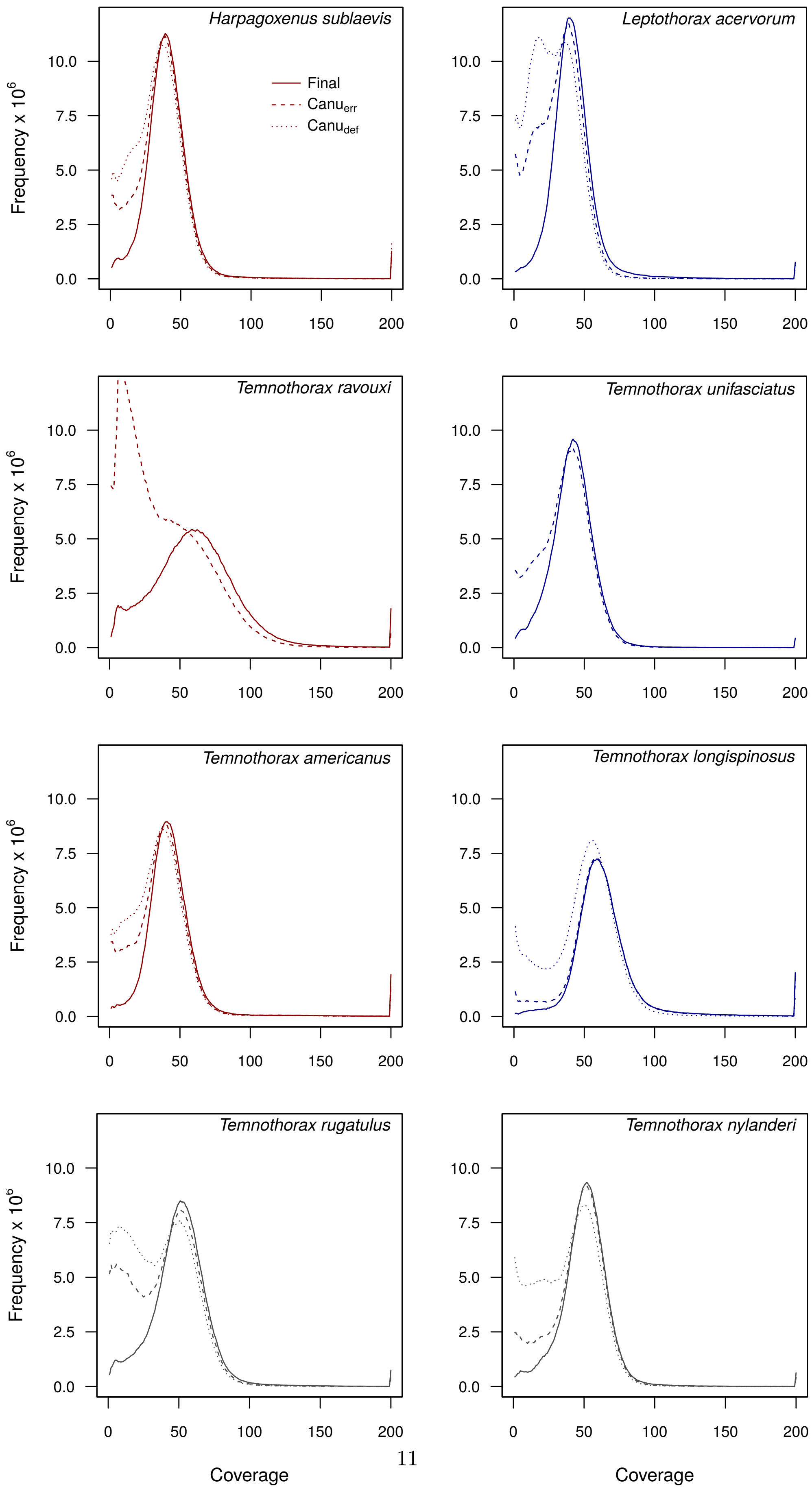

Figure S1: Read depth distribution of the eight genome assemblies.

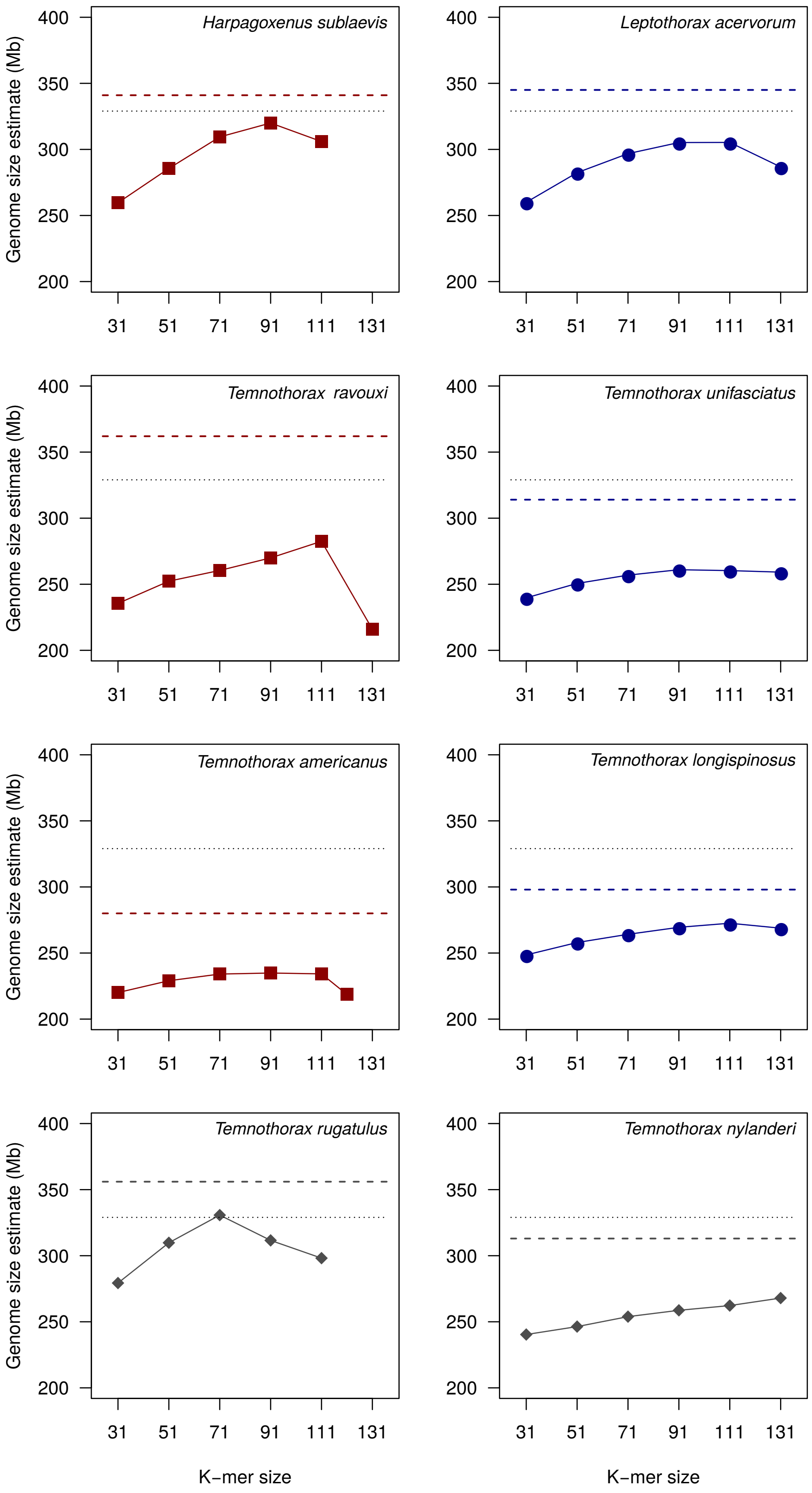

Figure S2: K-mer based genome size estimates. Short dashed, black line represents the average genome size of Myrmicinae [Tsutsui et al., 2008], long-dashed, colored line represents the final, PacBio assembly sizes.

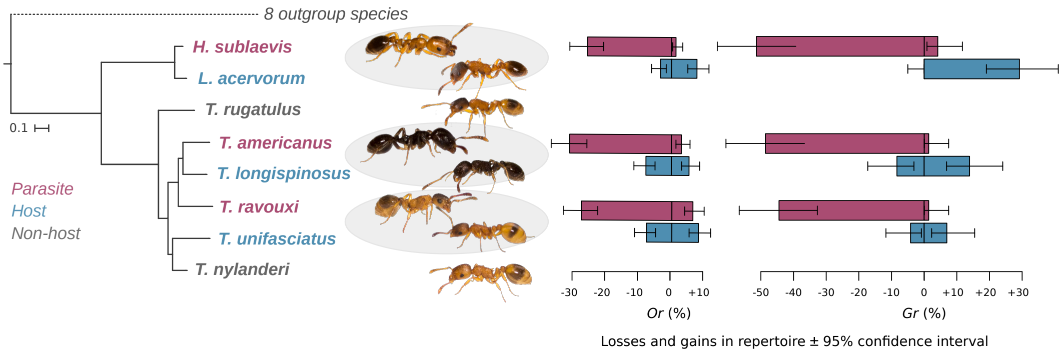

Figure S3: Phylogeny of the focal species in this study. Gray ellipses highlight the three independent parasite-host pairs studied. Figure based on Prebus 2017. Outgroup species are listed in S11. The dotted line to the 8 outgroup species is not to scale. Bars represent the percentage of *Or*s and *Gr*s lost (< 0) and gained (> 0) in each of the slave-making ant species and their hosts. Pictures by Barbara Feldmeyer.

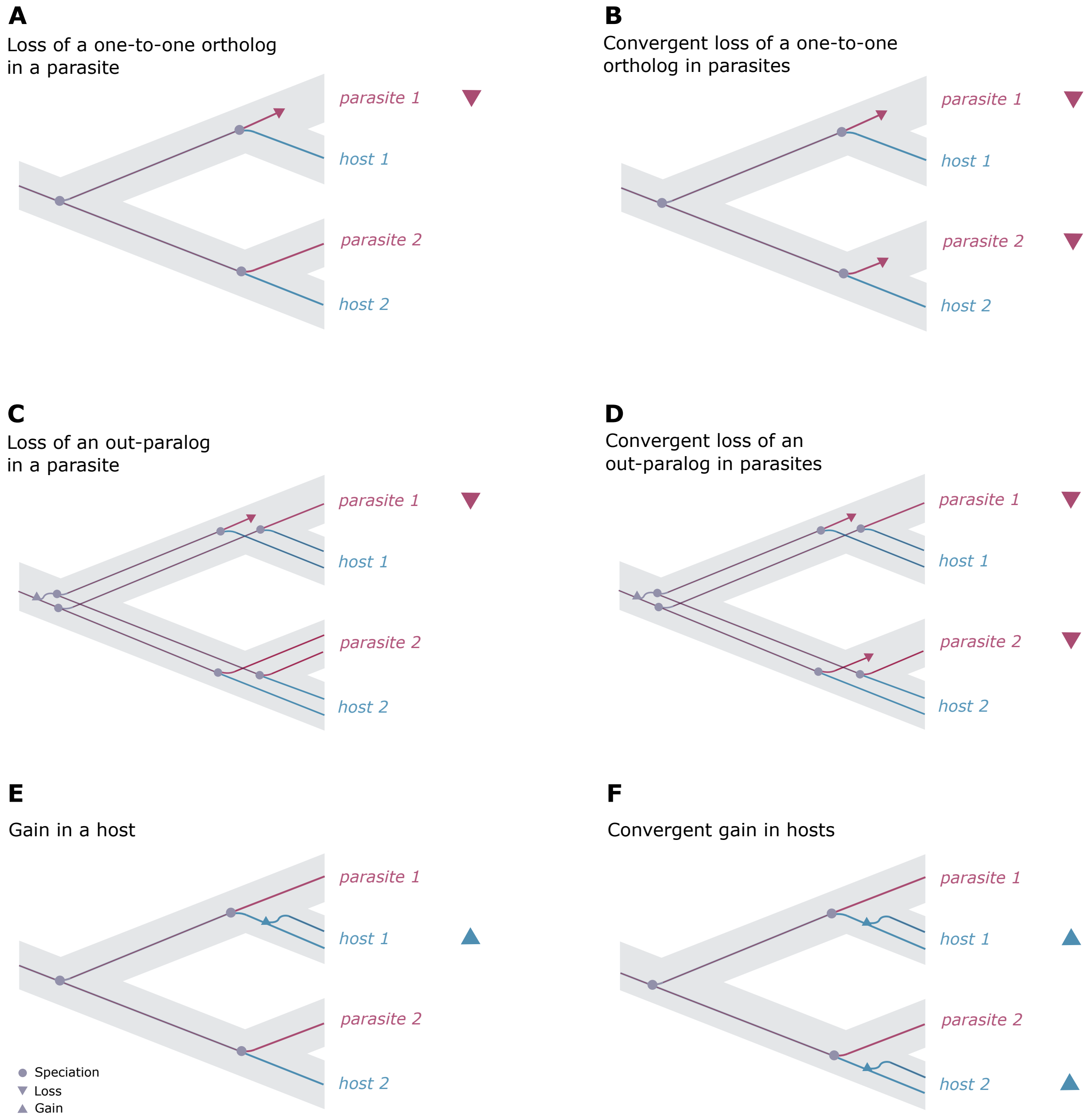

Figure S4: Conceptual diagrams of gene loss and gain in two slave-making ant (parasite) species and / or hosts. Left panel: loss and gain in individual species, right panel: convergent loss or gain across all parasites or hosts.

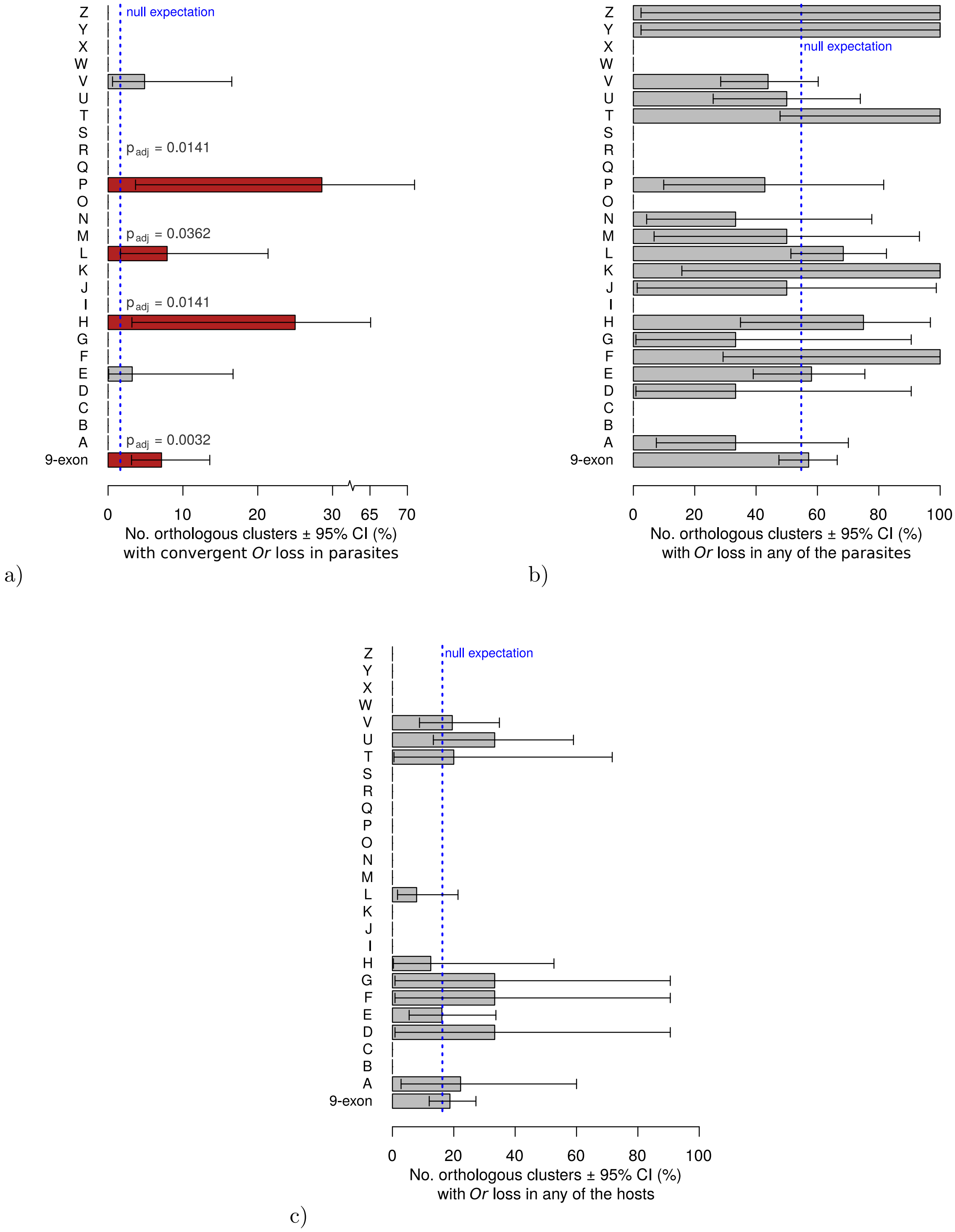

Figure S5: *Or* loss in relation to lifestyle.

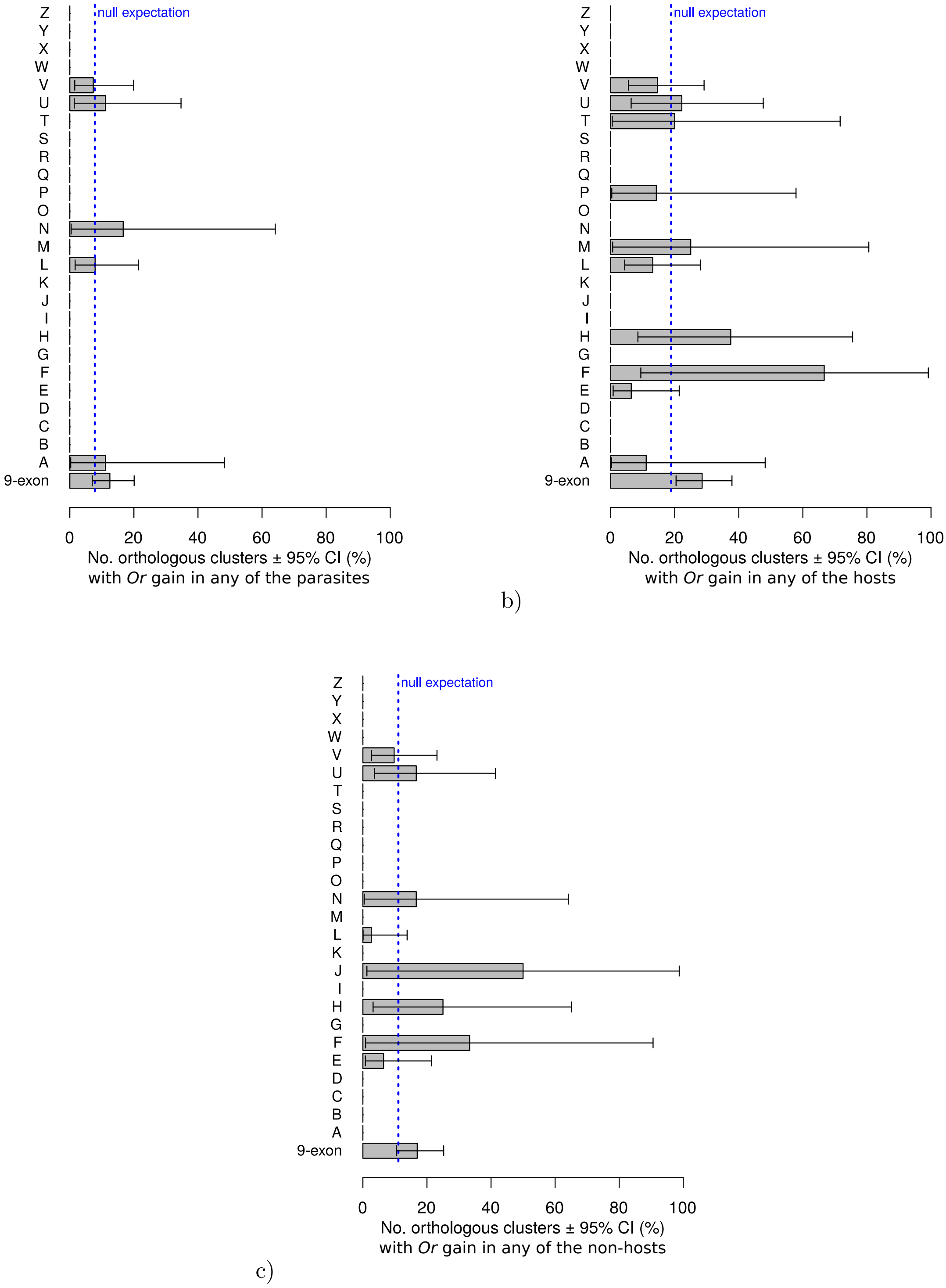

Figure S6: *Or* gain in relation to lifestyle.

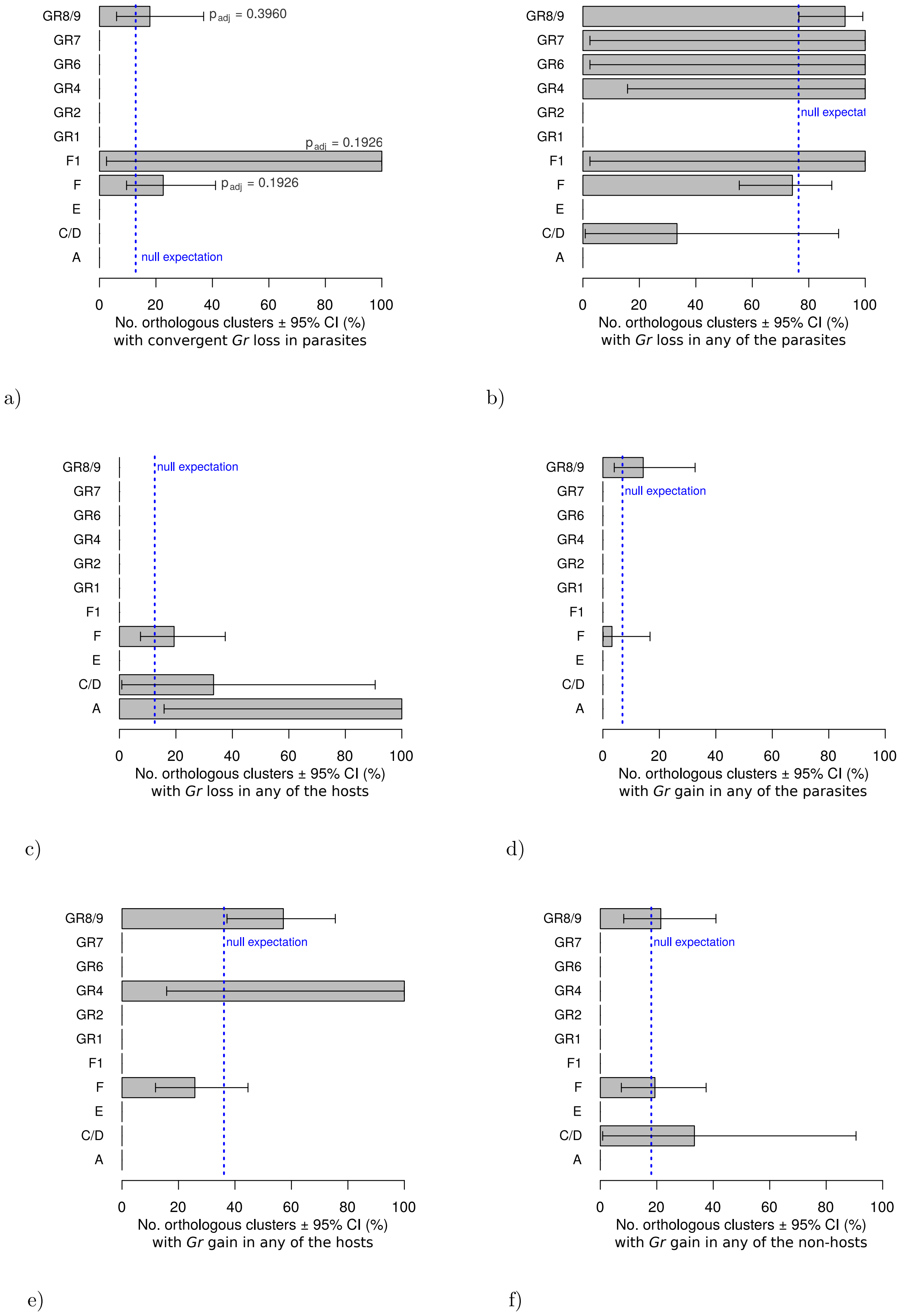

Figure S7: *Gr* gain and loss in relation to lifestyle.

References

E. Bao and L. Lan. HALC: High throughput algorithm for long read error correction. BMC Bioinformatics, 18(1), Apr. 2017. doi: 10.1186/s12859-017-1610-3. URL https://doi.org/10.1186/s12859-017-1610-3.

R. Bonasio, G. Zhang, C. Ye, N. S. Mutti, X. Fang, N. Qin, G. Donahue, P. Yang, Q. Li, C. Li, P. Zhang, Z. Huang, S. L. Berger, D. Reinberg, J. Wang, and J. Liebig. Genomic comparison of the ants camponotus floridanus and harpegnathos saltator. Science, 329(5995):1068–1071, Aug. 2010. doi: 10.1126/science.1192428. URL https://doi.org/10.1126/science.1192428.

M. J. Chaisson and G. Tesler. Mapping single molecule sequencing reads using basic local alignment with successive refinement (BLASR): application and theory. BMC Bioinformatics, 13(1), Sept. 2012. doi: 10.1186/1471-2105-13-238. URL https://doi.org/10.1186/1471-2105-13-238.

C. G. Elsik, A. Tayal, C. M. Diesh, D. R. Unni, M. L. Emery, H. N. Nguyen, and D. E. Hagen. Hymenoptera Genome Database: integrating genome annotations in HymenopteraMine. Nucleic Acids Research, 44(D1):D793–D800, Nov. 2015. doi: 10.1093/nar/gkv1208. URL https://doi.org/10.1093/nar/gkv1208.

A. Gurevich, V. Saveliev, N. Vyahhi, and G. Tesler. QUAST: quality assessment tool for genome assemblies. Bioinformatics, 29(8):1072–1075, Feb. 2013. doi: 10.1093/bioinformatics/btt086. URL https://doi.org/10.1093/bioinformatics/btt086.

J. Huerta-Cepas, F. Serra, and P. Bork. ETE 3: Reconstruction, analysis, and visualization of phylogenomic data. Molecular Biology and Evolution, 33(6):1635–1638, Feb. 2016. doi: 10.1093/molbev/msw046. URL https://doi.org/10.1093/molbev/msw046.

K. Katoh and D. M. Standley. MAFFT multiple sequence alignment software version 7: Improvements in performance and usability. Molecular Biology and Evolution, 30(4):772–780, Jan. 2013. doi: 10.1093/molbev/mst010. URL https://doi.org/10.1093/molbev/mst010.

S. Koren, B. P. Walenz, K. Berlin, J. R. Miller, N. H. Bergman, and A. M. Phillippy. Canu: scalable and accurate long-read assembly via adaptive k-mer weighting and repeat separation. Genome Research, 27(5):722–736, Mar. 2017. doi: 10.1101/gr.215087.116. URL https://doi.org/10.1101/gr.215087.116.

K.-K. Lam, K. LaButti, A. Khalak, and D. Tse. FinisherSC: a repeat-aware tool for upgrading *de novo* assembly using long reads. Bioinformatics, 31(19):3207–3209, June 2015. doi: 10.1093/bioinformatics/btv280. URL https://doi.org/10.1093/bioinformatics/btv280.

B. Langmead and S. L. Salzberg. Fast gapped-read alignment with Bowtie 2. Nature Methods, 9(4):357–359, Mar. 2012. doi: 10.1038/nmeth.1923. URL https://doi.org/10.1038/nmeth.1923.

H. Li. Minimap2: pairwise alignment for nucleotide sequences. Bioinformatics, 34(18):3094–3100, May 2018. doi: 10.1093/bioinformatics/bty191. URL https://doi.org/10.1093/bioinformatics/bty191.

S. Nygaard, G. Zhang, M. Schiøtt, C. Li, Y. Wurm, H. Hu, J. Zhou, L. Ji, F. Qiu, M. Rasmussen, H. Pan, F. Hauser, A. Krogh, C. J. Grimmelikhuijzen, J. Wang, and J. J. Boomsma. The genome of the leaf-cutting antAcromyrmex echinatiorsuggests key adaptations to advanced social life and fungus farming. Genome Research, 21(8):1339–1348, June 2011. doi: 10.1101/gr.121392.111. URL https://doi.org/10.1101/gr.121392.111.

M. Prebus. Insights into the evolution, biogeography and natural history of the acorn ants, genus *Temnothorax* Mayr (Hymenoptera: Formicidae). BMC Evolutionary Biology, 17(1), Dec. 2017. doi: 10.1186/s12862-017-1095-8. URL https://doi.org/10.1186/s12862-017-1095-8.

M. N. Price, P. S. Dehal, and A. P. Arkin. FastTree 2 – approximately maximum-likelihood trees for large alignments. PLoS ONE, 5(3):e9490, Mar. 2010. doi: 10.1371/journal.pone.0009490. URL https://doi.org/10.1371/journal.pone.0009490.

M. J. Roach, S. A. Schmidt, and A. R. Borneman. Purge Haplotigs: allelic contig reassignment for third-gen diploid genome assemblies. BMC Bioinformatics, 19(460), Nov. 2018. doi: 10.1186/s12859-018-2485-7. URL https://doi.org/10.1186/s12859-018-2485-7.

L. Salmela and E. Rivals. LoRDEC: accurate and efficient long read error correction. Bioinformatics, 30(24):3506–3514, Aug. 2014. doi: 10.1093/bioinformatics/btu538. URL https://doi.org/10.1093/bioinformatics/btu538.

L. Schrader, J. W. Kim, D. Ence, A. Zimin, A. Klein, K. Wyschetzki, T. Weichselgartner, C. Kemena, J. Stökl, E. Schultner, Y. Wurm, C. D. Smith, M. Yandell, J. Heinze, J. Gadau, and J. Oettler. Transposable element islands facilitate adaptation to novel environments in an invasive species. Nature Communications, 5(1), Dec. 2014. doi: 10.1038/ncomms6495. URL https://doi.org/10.1038/ncomms6495.

F. A. Simão, R. M. Waterhouse, P. Ioannidis, E. V. Kriventseva, and E. M. Zdobnov. BUSCO: assessing genome assembly and annotation completeness with single-copy orthologs. Bioinformatics, 31(19):3210–3212, June 2015. doi: 10.1093/bioinformatics/btv351. URL https://doi.org/10.1093/bioinformatics/btv351.

C. D. Smith, A. Zimin, C. Holt, E. Abouheif, R. Benton, E. Cash, V. Croset, C. R. Currie, E. Elhaik, C. G. Elsik, et al. Draft genome of the globally widespread and invasive argentine ant (linepithema humile). Proceedings of the National Academy of Sciences, 108(14):5673–5678, 2011a.

C. R. Smith, C. D. Smith, H. M. Robertson, M. Helmkampf, A. Zimin, M. Yandell, C. Holt, H. Hu, E. Abouheif, R. Benton, et al. Draft genome of the red harvester ant pogonomyrmex barbatus. Proceedings of the National Academy of Sciences, 108(14):5667–5672, 2011b.

G. Suen, C. Teiling, L. Li, C. Holt, E. Abouheif, E. Bornberg-Bauer, P. Bouffard, E. J. Caldera, E. Cash, A. Cavanaugh, O. Denas, E. Elhaik, M.-J. Favé, J. Gadau, J. D. Gibson, D. Graur, K. J. Grubbs, D. E. Hagen, T. T. Harkins, M. Helmkampf, H. Hu, B. R. Johnson, J. Kim, S. E. Marsh, J. A. Moeller, M. C. Muñoz-Torres, M. C. Murphy, M. C. Naughton, S. Nigam, R. Overson, R. Rajakumar, J. T. Reese, J. J. Scott, C. R. Smith, S. Tao, N. D. Tsutsui, L. Viljakainen, L. Wissler, M. D. Yandell, F. Zimmer, J. Taylor, S. C. Slater, S. W. Clifton, W. C. Warren, C. G. Elsik, C. D. Smith, G. M. Weinstock, N. M. Gerardo, and C. R. Currie. The genome sequence of the leaf-cutter ant atta cephalotes reveals insights into its obligate symbiotic lifestyle. PLoS Genetics, 7(2):e1002007, Feb. 2011. doi: 10.1371/journal.pgen.1002007. URL https://doi.org/10.1371/journal.pgen.1002007.

N. D. Tsutsui, A. V. Suarez, J. C. Spagna, and J. S. Johnston. The evolution of genome size in ants. BMC Evolutionary Biology, 8(1):64, 2008. doi: 10.1186/1471-2148-8-64. URL https://doi.org/10.1186/1471-2148-8-64.

B. J. Walker, T. Abeel, T. Shea, M. Priest, A. Abouelliel, S. Sakthikumar, C. A. Cuomo, Q. Zeng, J. Wortman, S. K. Young, and A. M. Earl. Pilon: An integrated tool for comprehensive microbial variant detection and genome assembly improvement. PLoS ONE, 9(11):e112963, Nov. 2014. doi: 10.1371/journal.pone.0112963. URL https://doi.org/10.1371/journal.pone.0112963.

Y. Wurm, J. Wang, O. Riba-Grognuz, M. Corona, S. Nygaard, B. G. Hunt, K. K. Ingram, L. Falquet, M. Nipitwattanaphon, D. Gotzek, M. B. Dijkstra, J. Oettler, F. Comtesse, C.-J. Shih, W.-J. Wu, C.-C. Yang, J. Thomas, E. Beaudoing, S. Pradervand, V. Flegel, E. D. Cook, R. Fabbretti, H. Stockinger, L. Long, W. G. Farmerie, J. Oakey, J. J. Boomsma, P. Pamilo, S. V. Yi, J. Heinze, M. A. D. Goodisman, L. Farinelli, K. Harshman, N. Hulo, L. Cerutti, I. Xenarios, D. Shoemaker, and L. Keller. The genome of the fire ant solenopsis invicta. Proceedings of the National Academy of Sciences, 108(14):5679–5684, Jan. 2011. doi: 10.1073/pnas.1009690108. URL https://doi.org/10.1073/pnas.1009690108.

A. V. Zimin, G. Marçais, D. Puiu, M. Roberts, S. L. Salzberg, and J. A. Yorke. The MaSuRCA genome assembler. Bioinformatics, 29(21):2669–2677, Aug. 2013. doi: 10.1093/bioinformatics/btt476. URL https://doi.org/10.1093/bioinformatics/btt476.
